# Uman-type neurofilament light antibodies are effective reagents for the imaging of neurodegeneration

**DOI:** 10.1093/braincomms/fcad067

**Published:** 2023-03-16

**Authors:** Gerry Shaw, Irina Madorsky, Ying Li, YongSheng Wang, Marda Jorgensen, Sabhya Rana, David D Fuller

**Affiliations:** EnCor Biotechnology Inc., Gainesville, FL 32608, USA; Department of Neuroscience, University of Florida College of Medicine, Gainesville, FL 32610, USA; McKnight Brain Institute, University of Florida, Gainesville, FL 32610, USA; EnCor Biotechnology Inc., Gainesville, FL 32608, USA; EnCor Biotechnology Inc., Gainesville, FL 32608, USA; EnCor Biotechnology Inc., Gainesville, FL 32608, USA; EnCor Biotechnology Inc., Gainesville, FL 32608, USA; McKnight Brain Institute, University of Florida, Gainesville, FL 32610, USA; Department of Physical Therapy, University of Florida, Gainesville, FL 32610, USA; Breathing Research and Therapeutics Center, University of Florida, Gainesville, FL 32610, USA; McKnight Brain Institute, University of Florida, Gainesville, FL 32610, USA; Department of Physical Therapy, University of Florida, Gainesville, FL 32610, USA; Breathing Research and Therapeutics Center, University of Florida, Gainesville, FL 32610, USA

**Keywords:** neurofilament, biomarker, NF-L, neurodegeneration, ELISA

## Abstract

Recent work shows that certain antibody-based assays for the neurofilament light chain detect informative signals in the CSF and blood of human and animals affected by a variety of CNS injury and disease states. Much of this work has been performed using two mouse monoclonal antibodies to neurofilament light, UD1 and UD2, also known as Clones 2.1 and 47.3, respectively. These are the essential components of the Uman Diagnostics Neurofilament-Light™ ELISA kit, the Quanterix Simoa™ bead-based assay and others. We show that both antibodies bind to neighbouring epitopes in a short, conserved and unusual peptide in the centre of the neurofilament light Coil 2 segment of the ‘rod’ domain. We also describe a surprising and useful feature of Uman and similar reagents. While other well-characterized neurofilament antibodies generally show robust staining of countless cells and processes in CNS sections from healthy rats, both Uman antibodies reveal only a minor subset of profiles, presumably spontaneously degenerating or degenerated neurons and their processes. However, following experimental mid-cervical spinal cord injuries to rats, both Uman antibodies recognize numerous profiles in fibre tracts damaged by the injury administered. These profiles were typically swollen, beaded, discontinuous or sinusoidal as expected for degenerating and degenerated processes. We also found that several antibodies to the C-terminal ‘tail’ region of the neurofilament light protein bind undamaged axonal profiles but fail to recognize the Uman-positive material. The unmasking of the Uman epitopes and the loss of the neurofilament light tail epitopes can be mimicked by treating sections from healthy animals with proteases suggesting that the immunostaining changes we discovered are due to neurodegeneration-induced proteolysis. We have also generated a novel panel of monoclonal and polyclonal antibodies directed against the Uman epitopes that have degeneration-specific staining properties identical to the Uman reagents. Using these, we show that the region to which the Uman reagents bind contains further hidden epitopes distinct from those recognized by the two Uman reagents. We speculate that the Uman-type epitopes are part of a binding region important for higher order neurofilament assembly. The work provides important insights into the properties of the Uman assay, describes novel and useful properties of Uman-type and neurofilament light tail-binding antibodies and provides a hypothesis relevant to further understanding of neurofilament assembly.

## Teaser

Shaw *et al.* show that both monoclonal antibodies used in the Uman Neurofilament-Light™ and related biomarker assays bind epitopes within the neurofilament light chain hidden in healthy neurons but exposed to neurodegeneration. They then show that a panel of novel Uman-type antibodies is useful immunocytochemical markers of neurodegeneration.

## Graphical Abstract

**Graphical Abstract:**
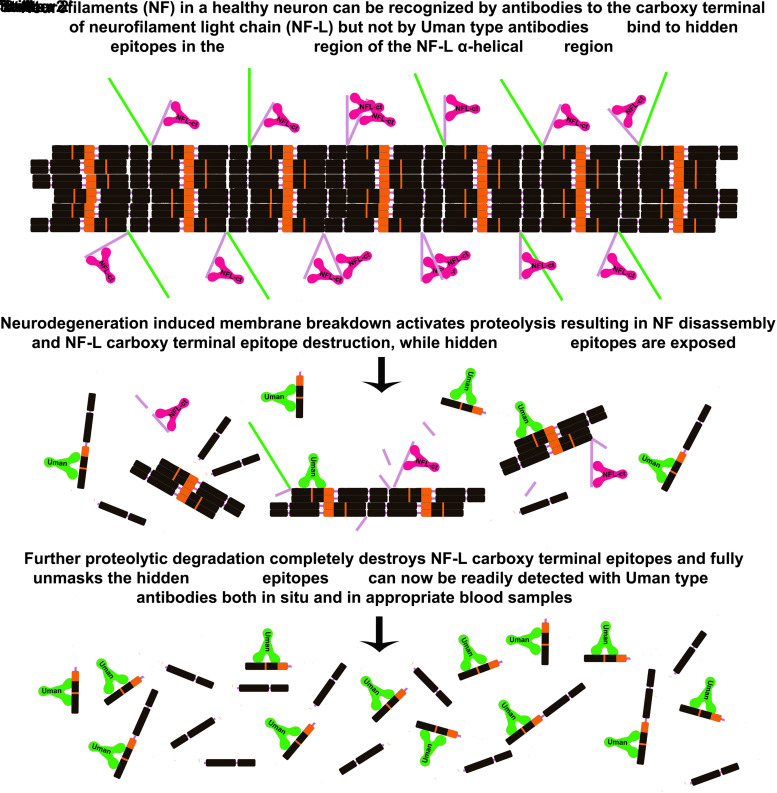

## Introduction

A major focus of recent research has been the identification of potential CNS damage and disease biomarkers that may be used diagnostically, prognostically or for monitoring responses to treatment. Axons are uniquely sensitive to damage and disease^1,2^ and contain large amounts of neurofilaments (NFs). Accordingly, the detection of proteins or protein fragments derived from NFs in blood or other fluids should provide information about ongoing axonal loss. Previously, we pioneered the use of the phosphorylated axonal form of the NF heavy chain [pNF-H, Human Genome Nomenclature Committee (HGNC) name NEFH] as a viable blood biomarker of axonal damage and degeneration.^3,4^ Recently, much interest has focused on another NF subunit, the light or low molecular weight NF protein, NF light polypeptide (NF-L), particularly since it can also be sensitively detected in patient blood.^5–13^ NF-L, also known as Nfl or by the HGNC name NEFL, is, like pNF-H, highly abundant, neuron-specific and heavily concentrated in axons.

**Figure 1:**
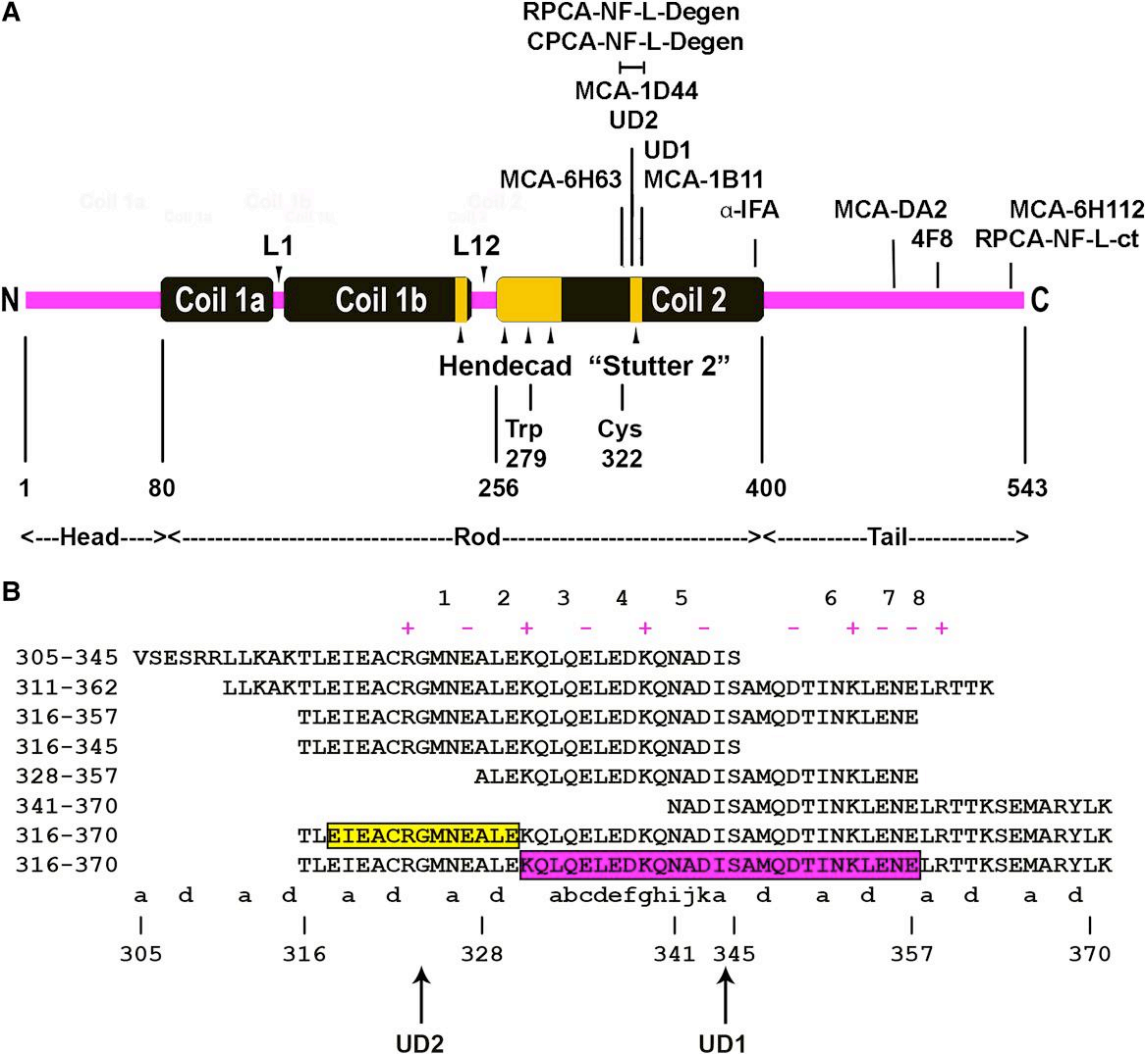
**Diagrams of NF-L structure, landmarks and epitopes.** (**A**) Domain organization and epitope map of human NF-L protein. The yellow blocks in Coil 1b and Coil 2 indicate the location of 11 amino acid ‘hendecad’ sequences, one of which is referred to here as ‘Stutter 2’. Location of landmarks and relevant antibody epitopes are based on the human NF-L protein sequence. (**B**) Amino acid sequences including the Uman epitopes and the various peptides used. The core of the UD2 epitope is outlined, as is the extended peptide essential for UD1 binding. The ‘a’ and ‘d’ positions of the hydrophobic heptad repeat are identified below the sequences. The region of the ‘Stutter 2’ where the normal heptad repeat is replaced by a hendecad sequence is indicated in the 333-343 region, with the addition of ‘h’, ‘i’, ‘j’ and ‘k’ amino acids. The charged i, i + 4 type residues are indicated with appropriate + and − signs above the sequences, and each instance is numbered in magenta. The C-terminal cysteine residues added to all peptides are omitted.

NF-L and pNF-H are members of the intermediate or 10 nm filament family of proteins consisting of over 60 gene products in humans that include other NF subunits, glial fibrillary acidic protein (GFAP), vimentin, the epithelial keratins and the nuclear lamins. All are based on a tripartite structure deduced from their amino acid sequence,^14^ now confirmed by structural studies.^15^ An N-terminal globular ‘head’ region is followed by a central α-helical ‘rod’ region followed by a C-terminal ‘tail’ segment (Fig. 1A). The structure of the central rod region is mostly a continuous α-helix except for two short non-helical linkers called L1 and L2. The α-helical regions are primarily characterized by the hydrophobic ‘heptad’ repeat.^16^ Sets of seven amino acids can be labelled from ‘a’ to ‘g’ repetitively down the amino acid sequence, and in the correct register, the a and d positions are typically hydrophobic. In all intermediate filament subunits, each α-helical region forms a stable parallel dimer with another similar polypeptide due to the hydrophobic a and d amino acids in one α-helix interacting with those in another similar α-helix allowing the two α-helices to wrap around each other forming an α-helical coiled coil.^14^ The Coil 2 sequence contains three regions in which the heptad pattern breaks down, being replaced by 11 amino acid ‘hendecad’ ^17^ repeats that do not perturb the α-helical coiled-coil structure.^15^ The C-terminal of Coil 1b and the N-terminal region of Coil 2 have one and three of these, respectively, and a single one is seen in the middle of Coil 2, known as ‘Stutter 2’ for historical reasons (Fig. 1A). Coiled-coil dimers associate laterally and end to end with other similar dimers to produce tetramers and higher order structures, a series of interactions that will finally produce an assembled 10 nm diameter filament.^18^

In 2002, Norgren *et al*.^19^ described a panel of mouse monoclonal antibodies made against purified mammalian spinal cord NF-L, two of which, Clones 2.1 and 47.3, proved to sensitively detect NF-L by ELISA.^20,21^ These two antibodies later became the key reagents in a commercial assay produced by Uman Diagnostics (Umeå, Sweden), the NF-Light™ ELISA, now sold by Quanterix (Billerica, MA). Quanterix also markets the significantly more sensitive single molecule array (Simoa™) bead-based platform using these antibodies which are also used in other assays. The 2.1 antibody serves as the NF-L detection reagent and 47.3 as the capture reagent, which in the Uman kit are referred to as UD1 and UD2, respectively. Most of the recent rapidly growing interest in NF-L as a biomarker of CNS damage and disease is a result of the efficiency of these two antibodies in the Uman and other assays particularly as applied to human blood samples.^5–13^

The original study of Norgren *et al*.^19^ showed that both Uman antibodies bound a chymotryptic fragment of NF-L identified as the entire ∼320 amino acid α-helical rod region (Fig. 1A). Lower molecular weight chymotryptic fragments were bound identically by the two antibodies, suggesting that the relevant epitopes were close together although the identity of these fragments was not determined. Neither antibody inhibited the binding of the other to NF-L, suggesting that they bind to distinct and non-overlapping epitopes. Attempts at epitope mapping with nested peptides and by phage display were unsuccessful. Fully understanding a biomarker assay requires knowledge of exactly what it is detecting. Here, we reveal the location of the epitopes for both Uman antibodies and show that surprisingly neither antibody stains NFs in healthy cells but both strongly recognize degenerating and degenerated neuronal material. These findings stimulated us to generate a panel of novel NF-L antibodies to the Uman epitopic region that share these interesting neurodegeneration-specific staining properties and that are briefly described here for the first time.

## Materials and methods

### Recombinant constructs

We generated full-length human NF-L, human NF-L rod region and human NF-L Coil 2 region, based on the sequence in NP_006149.2, with DNA sequences from GenScript (Piscataway, NJ). Codon-optimized DNAs were inserted into the pET30a(+) vector that adds an N-terminal His-tag, S-tag and some other sequences, a total of 52 extra amino acids. Expressed constructs were purified in 6 M urea in 10 mM phosphate buffer pH = 8.0 using the Nickel affinity of the N-terminal His-tag by standard methods.

### Protein chemistry

Cleavage at tryptophan was performed with 3-bromo-3-methyl-2-((2-nitrophenyl)thio)-3*H*-indole (BNPS-Skatole), and cleavage at cysteine residues was done using 2-nitro-5-thiocyanobenzoate (NTCB) as described.^22,23^ Both chemicals were obtained from Sigma-Aldrich (St. Louis, MO).

### Antibodies

Supplementary Table 1 lists relevant details of all antibodies used in this article. For certain experiments, 1 mg amounts of purified MCA-DA2, MCA-1B11 or MCA-6H63 were directly labelled with the fluorescent dyes Alexa Fluor® 488 or Alexa Fluor® 647 using Invitrogen/Life Technologies Protein Labeling Kits and following the manufacturer’s protocol (cat# A10235, A20173).

### Peptides

We designed 36 20-amino acid peptides each overlapping the next by five amino acids based on the entire human NF-L sequence in NP_006149.2 (Sigma-Aldrich). Peptides in 0.5 mg amounts were diluted in 50% acetonitrile/50% PBS to a concentration of 2.5 mg/mL and 2 µL of each peptide was arrayed in a 96-well plate along with 100 µL of the antibody to be tested at 1 µg/mL in PBS plus 0.1% non-fat milk. The final concentration of peptide was ∼21.7 µM, while the antibody concentration was about 6.6 nM, a 3260-fold molar peptide/antibody excess. After 10 min, the mix was transferred to an Immulon 4HBX plate previously coated overnight with 1 µg/mL recombinant full-length human NF-L in 50 mM sodium bicarbonate pH = 9.5 and then blocked with 5% non-fat milk in pH = 7.5 Tris-buffered saline plus 0.1% Tween 20 (TBSt). After 1 h at room temperature with shaking, the peptide/antibody mix plate was washed three times in TBSt, incubated for 1 h with 1 µg/mL goat anti-mouse alkaline phosphatase (Millipore Sigma Cat #AP124A) and washed again three times in TBSt, and signal was developed with p-nitrophenyl phosphate (Sigma Cat. #N2765), the colour reaction being quantified at 405 nm, corrected using a reference wavelength measurement at 595 nm.

Following the approximate localization of the Uman epitopes, Peptide 2.0 (Chantilly, VA, USA), synthesized human NF-L amino acids 316-370 with the addition of a C-terminal cysteine to facilitate coupling to carrier proteins. When appropriate, coupling was performed using Imject™ ovalbumin or Imject™ bovine serum albumin (BSA) following the manufacturer’s protocol (Thermo Fisher Cat# 77120 and 77110, respectively). Peptide 2.0 then synthesized human NF-L 305-345, 316-357, 316-345, 328-357 and 341-370, each with an additional C-terminal cysteine (Fig. 1B). For direct binding experiments, these were dissolved in TBSt and diluted to 5 mM, and 100 µL amounts were adsorbed onto Immulon 4HBX in 50 mM sodium bicarbonate pH = 9.5 overnight at 4°C and then blocked in 5% non-fat milk in TBSt. Antibody incubations and signal development were as described above.

For peptide competition experiments, we coated plates with 100 µL of full-length recombinant human NF-L at 0.5 µg/mL in 50 mM sodium bicarbonate pH = 9.5 and then blocked with 5% non-fat milk in TBSt. The various peptides were each diluted to 5 µM in TBSt, and 100 µL of each was added to the first well of a row of 10 wells on an ELISA plate; the remaining nine wells were filled with 50 µL of TBSt; 50 µL of the solution in the first well was serially diluted down the next eight wells, the 10th row serving as a blank, containing no peptide; 50 µL of antibody diluted to 1.0 µg/mL was added to each of the 10 wells, and the plate was incubated for 1 h at room temperature and further processed as described above. This experiment tests binding of 0.5 nM antibody to human NF-L in the presence of from 25 000 to 0 nM peptide.

Finally, GenScript synthesized 4 mg quantities of 35 25-amino acid peptides overlapping by one amino acid covering human NF-L 309-362, which were dissolved in 1 mL 50% distilled water/50% acetonitrile (Supplementary Fig. 3). Immulon 4HBX ELISA plates were coated with full-length NF-L as above, and 2 µL of each peptide was added sequentially to each of two wells. Control wells were incubated with 2 µL of 50% distilled water/50% acetonitrile; 100 µL of the relevant antibody was then added at 1 µg/mL and incubated for 1 h at room temperature with shaking. Washing, secondary antibody incubations and signal development were as described above.

### Spinal cord injury

A total of 10 12- to 20-week-old female Sprague–Dawley rats were obtained from Envigo (Hsd:Sprague Dawley® SD, Indianapolis, IN, USA). All procedures were approved by the Institutional Animal Care and Use Committee at the University of Florida (Protocol #201807438) and are in accordance with National Institutes of Health Guidelines. Animals were housed individually in cages under a 12-h light/dark cycle with *ad libitum* food and water. Animals were anaesthetized with ketamine (90 mg/kg) and xylazine (10 mg/kg) via intraperitoneal injection. Sufficient depth of anaesthesia was confirmed using a toe pinch resulting in lack of a change in heart rate, whisker twitch and hindlimb withdrawal. Anaesthesia was also continuously monitored during surgery and the animal was re-dosed with one-third of the initial ketamine/xylazine dose when needed. Body temperature was maintained with a water-recirculating heating pad. A dorsal incision was made from the base of the skull to the C6 region of the spine. Dorsal paravertebral muscles between C1 and C6 were incised and retracted. The procedures for C4 midline contusion have previously been described.^24,25^ Briefly, the rat was suspended by clamps on the C3 and C5 vertebrae. Rats were subjected to a single contusion injury at the midline, a force of 200 kdyn with 0-s dwell time, using a 2.5 mm diameter tip on an Infinite Horizon Impactor (Precision Systems and Instrumentation, Lexington, KY). For dorsal hemilesion, the dorsal aspect of the C4 spinal cord was sectioned using a spring scissor (Cat #15006-09, Fine Science Tools). To ensure correct depth of the lesion and consistency across animals, a 1 mm mark was made on the knife prior to lesioning. This process was repeated three times to ensure completeness of dorsal lesion. Subsequently, overlying muscles were sutured with sterile 4–0 Webcryl, and overlaying skin was closed using 9 mm wound clips. Animals were maintained on a heating pad until alert and awake and were monitored daily for signs of distress, dehydration and weight loss, with appropriate veterinary care as needed. Animals received buprenorphine (0.03 mg/kg, b.i.d.), meloxicam (1 mg/kg, q.d.), Baytril (5 mg/kg, q.d.) and lactated Ringer’s solution (10 mL/day, q.d.) for 48-h post-injury.

### Cell and tissue staining

Rats were anaesthetized with isofluorane, perfused with fresh 4% formaldehyde in PBS and tissues dissected out and stored overnight in fresh 4% formaldehyde at 4°C. They were transferred to PBS containing 15% sucrose for ∼2 days and then 30% sucrose for ∼2 days. Frozen sections were cut at 50 µM on a Leitz cryotome and stained using standard procedures.

Specimens were imaged on a Zeiss Axioskop 2 fitted with a Diagnostic Instruments RTke digital camera, a Keyence BZ-X810 microscope or an Olympus FV3000 confocal microscope.

### Protease experiments

Dried formaldehyde fixed 50 µM floating sections of uninjured rat spinal cord were rehydrated in PBS and then treated with 0.25% trypsin EDTA solution (Gibco cat# 25-200-056) or 20 µg/mL proteinase K (Thermo Fisher Cat# EO0492) for 10–120 min, washed in PBS to remove enzyme and fixed in 4% formaldehyde for 5 min, followed by regular immunostaining. Control sections were treated identically except for the omission of the enzyme. Enzyme-treated sections were imaged on a confocal microscope, and the exact settings optimized for these specimens were then used to image the relevant controls.

### Antibody generation

All procedures were performed according to the NIH Guide for the Care and Use of Experimental Animals. Twelve-week-old BALB/c mice were obtained from Jackson Labs (Bar Harbor, ME, USA) and were injected subcutaneously with 100 µL of 1 mg/mL proprietary recombinant immunogen containing NF-L 311-362 (Fig. 1B) mixed 1:1 with Freund’s complete adjuvant. Three weeks, later mice were boosted with the same immunogen in Freund’s incomplete adjuvant, and at 10 days, blood samples were screened by western blotting on rat spinal cord homogenates. Two mice with good immunoreactivity were boosted with the same immunogen, given a final intraperitoneal boost at 2 weeks, euthanized 3 days later and their spleens aseptically removed. Spleen cells were fused to PAI-O myeloma cells^26^ by standard methods, and fused cells were seeded into six 96-well culture dishes. Ten days later, hybridoma supernatants were screened by ELISA and western blotting on recombinant human NF-L, and promising clones were tested on sections of contused spinal cord. Useful clones were subcloned and characterized in detail.

### Statistical analysis

Data were initially collected and processed in Excel 16.6.4 (Microsoft Corp., Redmond, WA, USA) and further analysed, and figures were generated using GraphPad Prism 9.4.1 (GraphPad Software Inc., San Diego, CA, USA).

## Results

### Epitope mapping of Uman and other existing NF-L antibodies

A set of 20-amino acid–nested peptides based on the full-length sequence of human NF-L, in which each peptide overlapped the next by 5-amino acids, were used to test the inhibition of binding of Uman and various other available NF-L monoclonal antibodies to pure recombinant full-length human NF-L. UD2 binding was strongly inhibited by Peptide 22, amino acids 316-335 of the human NF-L sequence, which is just N-terminal to Stutter 2 in Coil 2 (Fig. 1A and Supplementary Fig. 1A and C). Peptide 21 showed weak inhibition suggesting that the overlapping 5-amino acids are an important part of the UD2 epitope, which we conclude is dependent on the peptide TLEIEACRGMNEALE, amino acids 316-330. The widely used antibody MCA-DA2 was strongly inhibited by Peptide 30, amino acids 436-455 of the human sequence and to a lesser degree by Peptide 31, amino acids 451-470 (Supplementary Fig. 1B and C). Antibody binding is therefore dependent on the sequence SYYTSHVQEEQIEVE, amino acids 441-455 in the ‘tail’ region. No convincing peptide inhibition of antibody binding to full-length human NF-L was seen with UD1 or several other NF-L antibodies tested.

**Figure 2:**
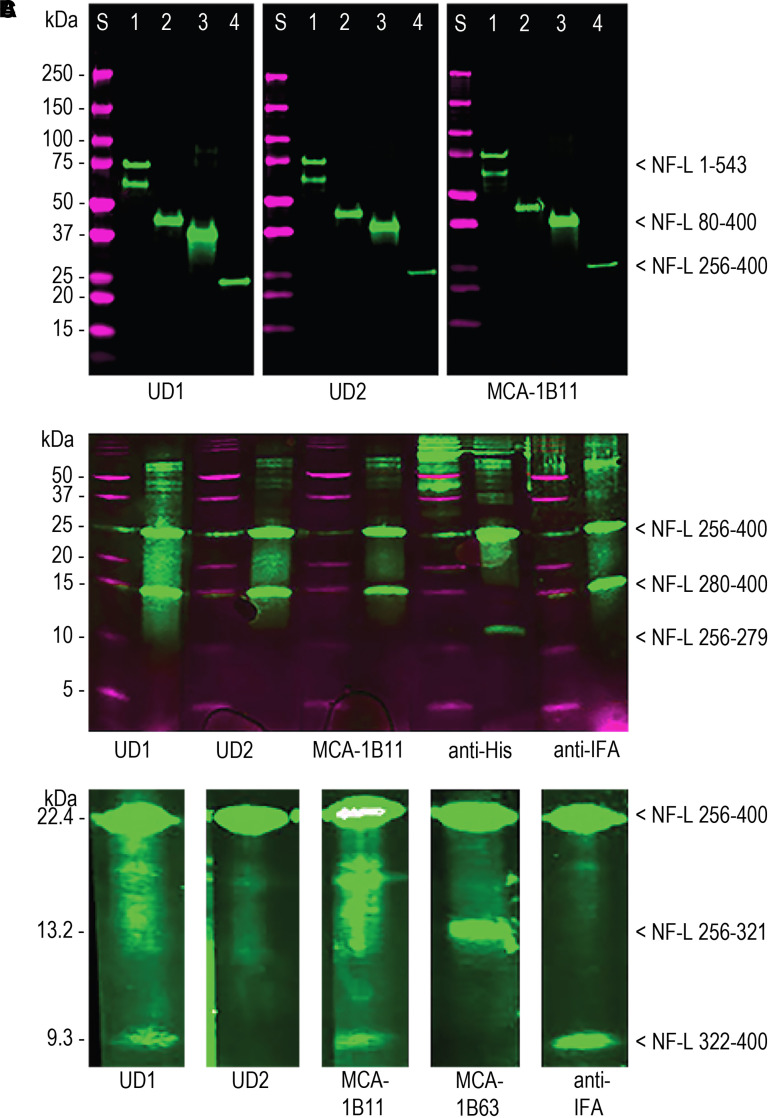
**Western blotting studies.** (**A)** Western blotting of Uman antibodies and MCA-1B11 on recombinant NF-L constructs. Lanes marked S are molecular weight standards of indicated size in magenta. In each case, Lane 1 is full-length human NF-L (1-543), Lane 2 is NF-L rod (80-400), Lane 3 is a proprietary immunogen containing NF-L 311-362 used to generate novel antibodies, and Lane 4 is NF-L Coil 2 (256-400). Original western blot images are in Supplementary Fig. 5 (left two panels) and Supplementary Fig. 6 (right panel) (**B**). Partial protein cleavage of NF-L 256-400 at tryptophan 279 produces two fragments of expected size 8.5 and 13.9 kDa. The N-terminal 8.5 kDa is identified by a His-tag antibody and the C-terminal 13.9 kDa by anti-IFA (see text). All three NF-L antibodies tested bind to the C-terminal 13.9 kDa band. Original western blot image is shown in Supplementary Fig. 7. (**C**) Partial cleavage of NF-L 256-400 at cysteine 322 produces an N-terminal 13.2 kDa fragment and a C-terminal 9.3 kDa fragment, identified using antibody to the vector-derived S-tag and anti-IFA. Both UD1 and MCA-1B11 bind the C-terminal 9.3 kDa fragment, while the epitope for UD2 is apparently destroyed. Original blots for UD1 and MCA-1B63 lanes are shown in Supplementary Fig. 8, while UD2, MCA-1B11 and anti-IFA lanes are shown in Supplementary Fig. 9.

Both Uman antibodies and MCA-1B11, an already available antibody, showed strong staining of native NF-L from a variety of species, on recombinant full-length human NF-L, on a rod construct (NF-L 80-400) and on a Coil 2 construct (NF-L 256-400 Fig. 1A and Fig. 2A). BNPS-skatole cleavage of NF-L 256-400 C-terminal to tryptophan 279 produces an N-terminal 8.5 kDa and a C-terminal 13.9 kDa fragment (Fig. 2B). The identity of the 13.9 kDa band was confirmed using anti-intermediate filament antigen (anti-IFA) antibody that binds a highly conserved epitope at the C-terminus of the rod region of all intermediate filament subunits, amino acids 380-400 in human NF-L.^27^ The identity of the N-terminal fragment was confirmed using an antibody to the vector-derived His-tag sequence. Both Uman antibodies and MCA-1B11 bound the 13.9 kDa C-terminal fragment mapping all three antibodies to within NF-L 280-400. NTCB cleavage of NF-L 256-400 N-terminal to cysteine 322 produces an N-terminal 13.2 kDa and a C-terminal 9.3 kDa fragment. The identity of these fragments was confirmed using anti-IFA and MCA-1B63 that bind the vector-derived N-terminal S-tag sequence (Fig. 2C). UD1 and MCA-1B11 bound the C-terminal 9.3 kDa fragment, while UD2 did not stain either fragment, although it recognized the uncleaved protein. This suggests that the UD2 epitope is destroyed by cleavage at cysteine 322 consistent with our finding that the 316-335 peptide including cysteine 322 inhibited binding of UD2 to NF-L.

The UD2 epitope is therefore associated with cysteine 322, while the UD1 and MCA-1B11 epitopes are C-terminal to but likely close to this. We synthesized human NF-L amino acids 316-370 with a C-terminal cysteine added (Fig. 1B). All three antibodies strongly bound this peptide coupled to ovalbumin and immobilized onto ELISA plates (Supplementary Fig. 1D), and all strongly bound to 316-370 peptide-ovalbumin and 316-370 peptide-BSA conjugates on western blots but not to unconjugated carrier proteins (Supplementary Fig. 1E). We then synthesized several smaller overlapping peptides (Fig. 1B). UD1 bound directly to 316-370 immobilized to ELISA plates and showed reduced binding to 316-357 and no binding to the other peptides **(**Supplementary Fig. 2A). UD2 strongly bound to NF-L 316-370 and to a lesser but significant degree to the three other peptides containing the 316-330 peptide (Supplementary Fig. 2B). We also tested the ability of each of the six peptides to inhibit antibody binding to recombinant human NF-L immobilized in an ELISA dish. The 316-370 peptide proved to be the most potent inhibitor of binding of both Uman reagents, suggesting that both are dependent on protein conformations not fully recapitulated by the shorter sequences. In line with the direct peptide-binding results for UD1, 316-357 showed reduced inhibition while the other peptides were ineffective (Supplementary Fig. 2C). Also consistent with previous data, UD2 binding was inhibited strongly by NF-L 316-370 and somewhat less efficiently by other peptides containing the 316-330 sequence while other peptides were ineffective (Supplementary Fig. 2D).

To further delineate the epitopes for these antibodies, we generated a set of 35 25-amino acid peptides overlapping by one amino acid and spanning the sequence from 309 to 362 (Supplementary Fig. 3C). We saw incomplete peptide inhibition of binding of UD1 to NF-L with all peptides from numbers 23 to 30, encompassing KQLQELEDKQNADISAMQDTINKLENE, NF-L 331-352 (Supplementary Fig. 3A). We conclude that the UD1 epitope is dependent on sequence within 331-352 but that strong binding requires more than any single 25-amino acid sequence (outlined in Fig. 1B and Supplementary Fig. 3A). Peptides that contain TLEIEACRGMNEALE, amino acids 316-330, all strongly inhibited UD2 binding to NF-L as expected (Supplementary Fig. 3B). However, inhibition disappeared with the loss of glutamic acid residue 318, allowing refinement of the core of the UD2 epitope to EIEACRGMNEALE, amino acids 318-330 (outlined in Fig. 1B).

### Immunostaining of control cells and tissues

**Figure 3:**
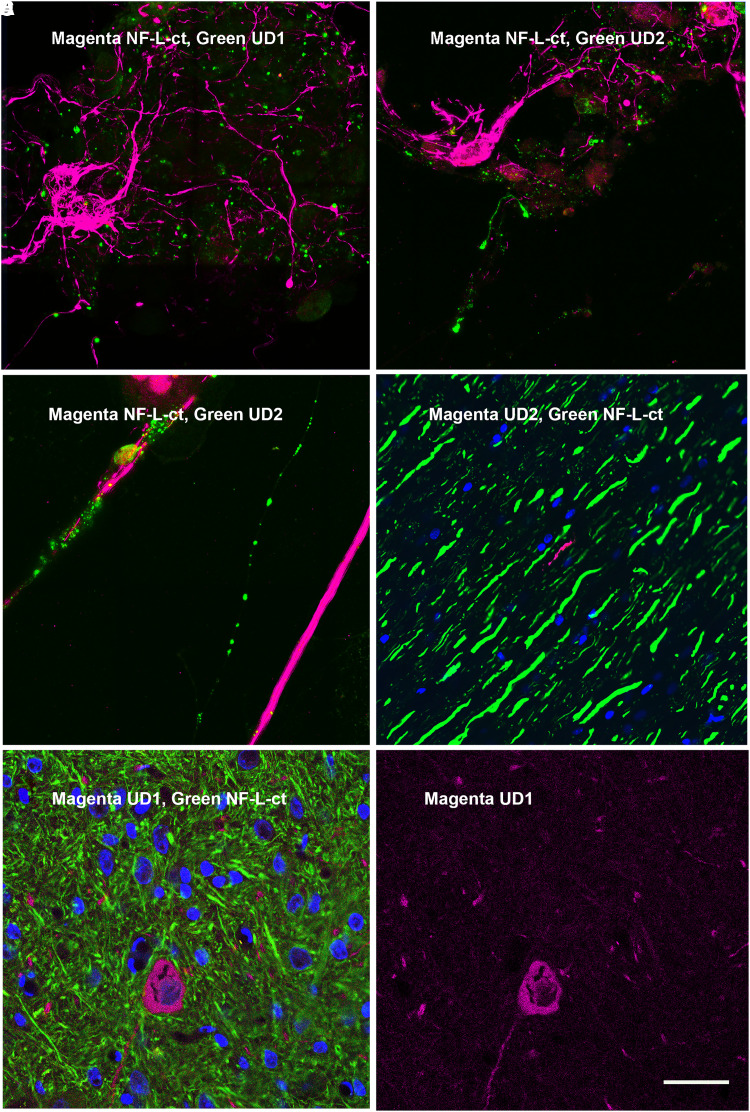
**Immunofluorescence of control cells and tissues.** (**A** and **B**) Seven- to 10-day neural cultures from E20 rats stained with UD1 (**A**) and UD2 (**B**) in green and both co-stained with RPCA-NF-L-ct in magenta. In both cases, the majority of the Uman-positive material is punctate and negative for RPCA-NF-L-ct, while the RPCA-NF-L-ct antibody stains fibrillar profiles with a typical neuronal morphology. **C** shows a region of a similar culture stained with UD2 in green and RPCA-NF-L-ct in magenta. The prominent linear array of Uman-positive globular material is suggestive of the remains of a degenerated process, while the RPCA-NF-L-ct–positive profile appears continuous and fibrillar. **D** shows a section of spinal cord from an uninjured rat stained with UD2 in magenta and RPCA-NF-L-ct in green. A single fibre negative for RPCA-NF-L-ct is revealed with the UD2 antibody. **E** and **F** show a region of brain stem from a control rat. One somewhat unhealthy looking neuron and associated processes is positive for UD2 (magenta) but not RPCA-NF-L-ct (green). **F** shows the same specimen with only the Uman antibody in magenta. The blue signal in **D** and **E** shows DNA staining with DAPI. Bar in image **F** = 50 µM, in **A**–**C** = 20 µM, in **D** = 40 µM and in **E** = 50µM.

We stained 7- to 10-day mixed E20 rat cortical neural cultures with both Uman antibodies in comparison with an affinity purified rabbit polyclonal antibody RPCA-NF-L-ct, raised against the rat NF-L C-terminal peptide, amino acids 515-543. To our surprise, both Uman antibodies failed to stain the majority of the clearly fibrillar neuronal profiles robustly stained with RPCA-NF-L-ct (Fig. 3A, UD1; Fig. 3B, UD2). Under higher magnification, we observed that staining with both antibodies was primarily punctate and most of the material reactive with either Uman antibody was negative for RPCA-NF-L-ct. A few linear profiles were stained with the Uman reagents which in the majority of cases showed no staining for RPCA-NF-L-ct (Fig. 3B). Punctate and globular Uman-positive profiles frequently appeared in discontinuous linear arrays that suggested that they may have originated from degenerated processes, in stark contrast to the well-defined RPCA-NF-L-ct–positive profiles (Fig. 3C). A significant amount of spontaneous neuronal death occurs in neural cultures similar to those used here.^28,29^

Both Uman reagents generally failed to stain normal adult rat spinal cord and brain sections in contrast to RPCA-NF-L-ct that robustly stained countless neurons and processes. We identified a few profiles in normal adult rat spinal cord that were positive for either UD1 or UD2 against a massive background of RPCA-NF-L-ct–positive Uman-negative material (Fig. 3D, UD2 shown). We also noted a few rare neuron cell bodies in brain that stained with one or other Uman reagent but not with RPCA-NF-L-ct (Fig. 3E and F). An occasional process or neuron that has undergone spontaneous degeneration is expected in normal CNS tissue.^30^

### Immunostaining of rat spinal cord tissue following SCI

**Figure 4:**
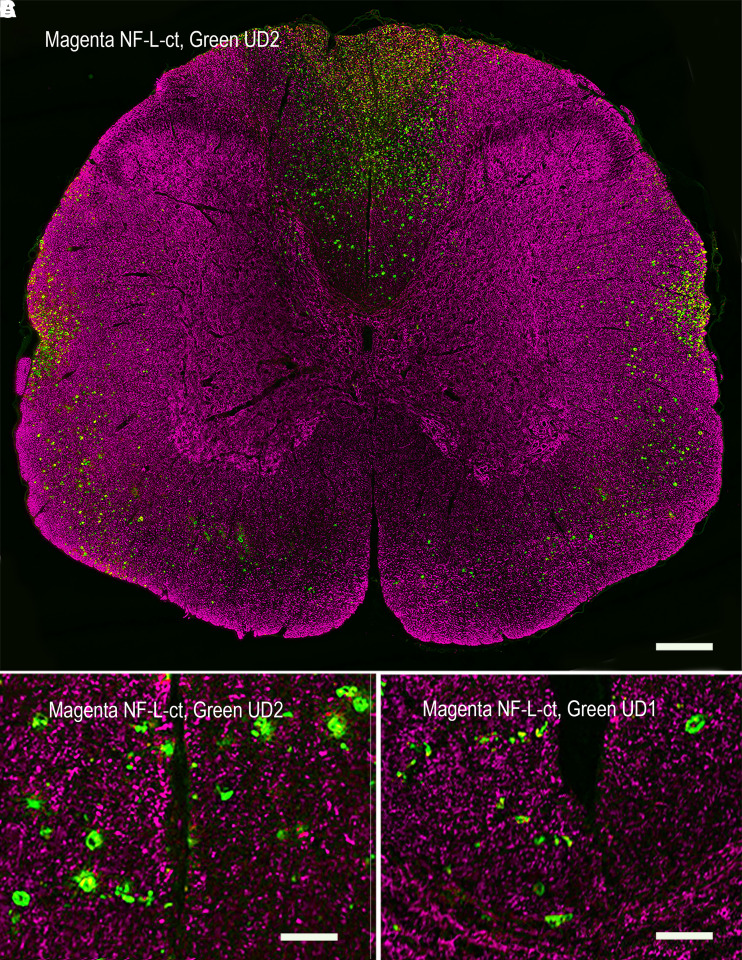
**Immunofluorescence of coronal sections from a rat given a spinal cord contusion 3 days previously.** **A** shows a coronal section stained for RPCA-NF-L-ct in magenta and UD2 in green. UD2-positive profiles are particularly obvious in the dorsal columns, corticospinal tracts and rubrospinal tracts, less abundant in the lateral and ventral funiculi and least abundant in the spinal cord grey matter. **B** shows a ×6 magnified view of the region just above the central canal of the same section shown in **A**, showing well-defined RPCA-NF-L-ct axonal profiles in magenta in comparison with the more diffuse and generally non-overlapping UD2 profiles in green. **C** shows a ×6 magnified view of the same region of a similar section stained with RPCA-NF-L-ct in magenta and UD1 in green. Bar in image **A** = 500 µM and in images **B** and **C** = 83 µM.

**Figure 5:**
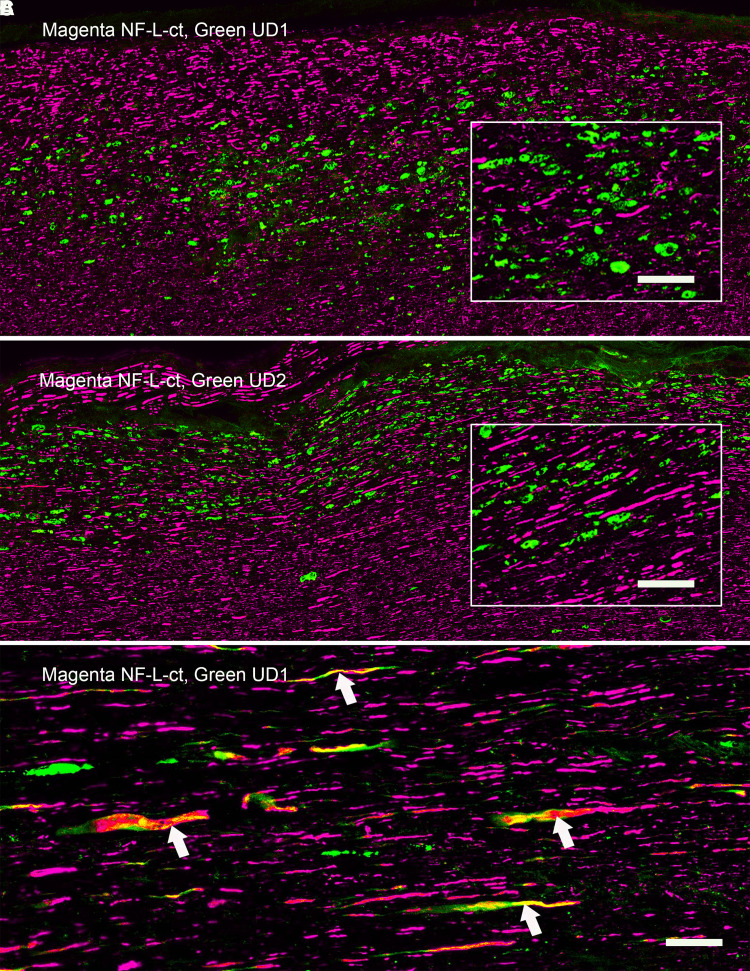
**Immunofluorescence of longitudinal sections from contused rat spinal cord 3 day after injury.** Lateral rubrospinal and neighbouring tracts some distance from the lesion. Linear arrays of profiles visualized with UD1 (**A**) and UD2 (**B**) shown in green are negative for RPCA-NF-L-ct shown in magenta. The RPCA-NFL-ct profiles are generally well-defined and continuous; the UD1 and UD2 profiles are mostly swollen, globular and discontinuous. Inserts show ×3 magnified sections of each image. **C** shows a region close to a contusion lesion and shows swollen and apparently degenerating axonal profiles positive for RPCA-NF-L-ct in magenta but also shows UD1 staining in green. Note a few the swollen and unhealthy looking profiles that show staining for both antibodies (arrowed). Bar in image **C** = 25 µM applicable to images **A**–**C** and bar in images **A** and **B** inserts = 8 µM.

**Figure 6:**
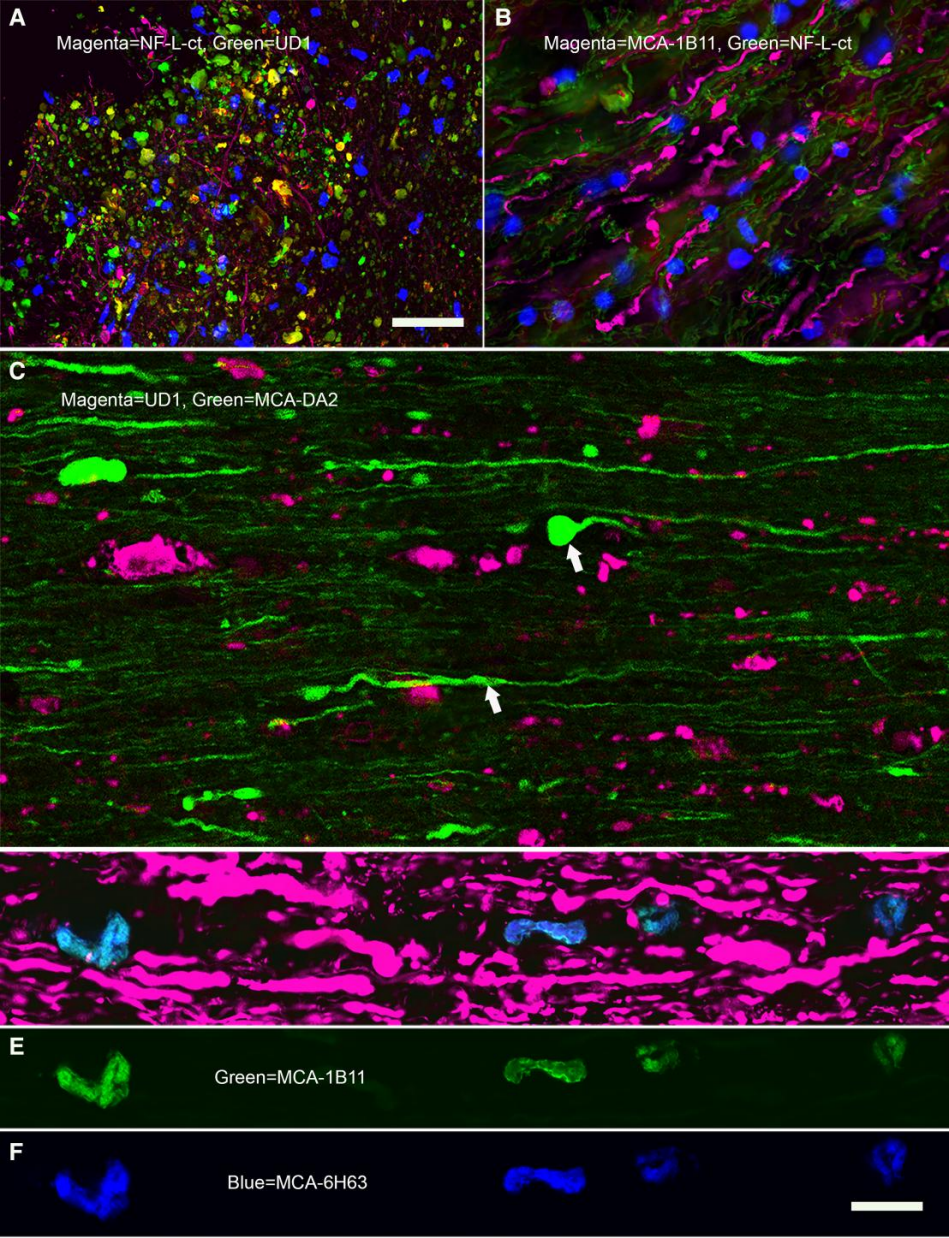
**Further immunofluorescence of contused rat spinal cord. A** shows a region at the lesion site of an animal given a contusion 3 days previously stained with RPCA-NF-L-ct in magenta and UD1 in green. Fibres positive for RPCA-NF-L-ct alone are visible amongst mostly globular profiles positive for both antibodies or only for UD1. **B** shows a region of adult mouse spinal cord that was allowed to sit at room temperature for 4 h prior to fixation. Sections were then incubated with RPCA-NF-L-ct in green and the Uman-type antibody MCA-1B11 in magenta. Note prominent beaded, discontinuous, sinusoidal and apparently helical MCA-1B11–positive profiles that are negative for RPCA-NF-L-ct, while RPCA-NF-L-ct staining is attenuated. **C** shows a longitudinal section from an SCI animal showing beaded, swollen and sinusoidal processes (arrowed) stained with MCA-DA2 directly labelled with Alexa Fluor® 488 and co-stained with UD1 in magenta. Like RPCA-NF-L-ct, MCA-DA2 does not stain most of the UD1-positive material. (**D**–**F**) A longitudinal section of spinal cord from an uninjured control rat was searched to find what we propose are spontaneously degenerating or degenerated nerve fibres as in Fig. 3D. **D** shows typical axonal profiles stained with RPCA-NF-L-ct in magenta. A linear group of globular profiles that were negative for RPCA-NF-L-ct but positive for MCA-1B11 (**E**, green) directly coupled to Alexa Fluor® 488 were also identically positive for MCA-6H63 coupled to Alexa Fluor® 647 (**F**, blue). MCA-1B11 and MCA-6H63 bind distinct hidden NF-L epitopes, building confidence that the objects stained are indeed degenerating or degenerated axons. Bar in image **D** = 10 µM, applicable to images **C**–**F**, and in image **A** = 20 µM, applicable to images **A** and **B**.

We evaluated spinal cord tissue from rats following C4 midline contusion or C4 dorsal hemilesion, expected to produce large numbers of degenerating and degenerated neurons and their processes.^24,25^ A series of both longitudinal and coronal sections from the injured region and flanking tissue were evaluated 1, 3 and 5 days after injury along with similar sections from untreated control animals. Control animals showed robust RPCA-NF-L-ct staining with virtually no staining with either Uman reagent. In marked and spectacular contrast, coronal sections processed 1, 3 or 5 days after injury show numerous profiles positive for both Uman antibodies that were mostly RPCA-NF-L-ct–negative, amongst a massive background of RPCA-NF-L-ct–positive, Uman-negative fibres (e.g. Figs 4 and 5). The Uman-positive profiles were seen in the dorsal columns, dorsal corticospinal tracts and lateral funiculi both rostral and caudal to the lesion site (Fig. 4A, UD2, green; RPCA-NF-L-ct, magenta). A few Uman-positive profiles were seen in other regions of the cord including in the grey matter. Under higher magnification, the profiles positive only for the Uman antibodies often appeared swollen, granular and diffuse, in contrast to the generally sharply defined axonal profiles positive for only RPCA-NF-L-ct (Fig. 4B, UD2; Fig. 4C, UD1). Longitudinal sections taken further from the lesion site revealed linear arrays of mostly swollen and discontinuous material negative for RPCA-NF-L-ct (Fig. 5A, UD1; Fig. 5B, UD2). These Uman-positive aggregates were visible >1 cm from the lesion site in dorsal and lateral spinal cord tracts at all time points post-injury. Closer to the lesion site, we saw a few examples of processes, usually swollen or sinusoidal, which stained for RPCA-NF-L-ct and one or other Uman antibody (Fig. 5C). Very close to the lesion site, we frequently saw spherical and elongated profiles that were positive for both RPCA-NF-L-ct and one or other Uman antibody (Fig. 6A).

We directly labelled the NF-L antibody MCA-DA2 with Alexa Fluor® 488, which allowed double label-immunofluorescence with each of the Uman reagents and this mouse antibody. The MCA-DA2 epitope is within the C-terminal tail of NF-L but distinct from the region recognized by RPCA-NF-L-ct (Fig. 1A). Like RPCA-NF-L-ct, MCA-DA2 recognizes both normal and some damaged neuronal processes, showing helical and typical retraction bulb endings close to the lesion site but does not stain most of the Uman-positive material (Fig. 6B). Two other NF-L tail antibodies, MCA-6H112 and 4F8,^32^ also bound RPCA-NF-L-ct–positive material and failed to bind the Uman-positive material (not shown).

We concluded that neuronal processes that were originally immunoreactive with certain NF-L tail antibodies lost this immunoreactivity while Uman epitopes concomitantly became unmasked. Some processes express both Uman and NF-L tail epitopes (Fig. 5C), but the relative paucity of these suggests that the transition from one state to the other is rather rapid, the degenerated material then expressing only the Uman epitopes. The obvious hypothesis to explain these findings is that degeneration activates proteases that remove or destroy the NF-L tail epitopes and concomitantly expose the Uman epitopes.

### Protease experiments

**Figure 7:**
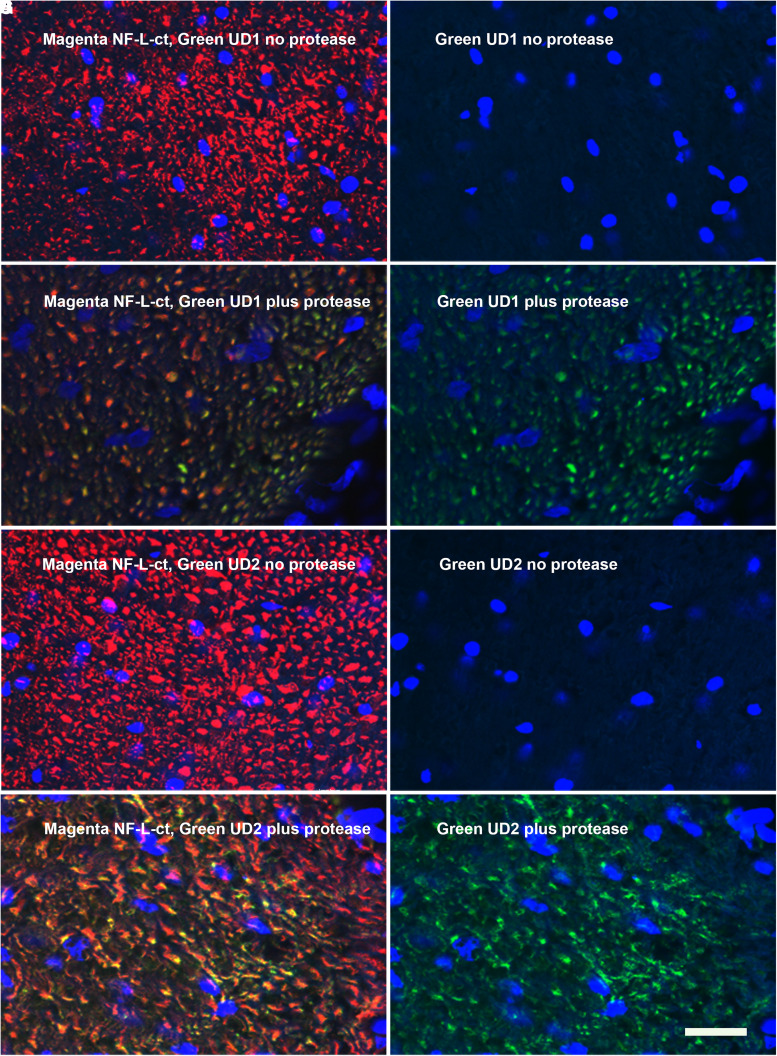
**Protease experiments.** Coronal sections of uninjured control rat spinal cords were treated with 0.25% trypsin or with buffer control lacking trypsin under exactly the same conditions. **A** shows a fibre tract after 10 min in control buffer then stained with RPCA-NF-L-ct in magenta and UD1 in green, while **B** shows just the green UD1 signal. **C** and **D** show a similar region incubated exactly as the section in **A** and **B** but with the addition of 0.25% trypsin. **C** and **D** were imaged on a confocal microscope, and identical laser and software settings were then used to images **A** and **B**. Following enzyme treatment, there is a weaker RPCA-NF-L-ct signal and a significant UD1 signal. **E** and **F** show similar control data for UD2 (green) co-stained with RPCA-NF-L-ct (magenta) on a similar section from an uninjured animal, and **G** and **H** show the staining of the same two antibodies following 10-min treatment in trypsin. The blue signal in all images is nuclear DNA revealed with DAPI. Bar in image **H** = 20 µM.

We incubated standard formalin-fixed coronal sections of uninjured rat spinal cord with proteinase K or trypsin for various times. Control tissue sections treated with appropriate protease buffer lacking enzyme showed strong staining with RPCA-NF-L-ct and essentially no staining with either Uman antibody (Fig. 7A, B, E and F). In sharp contrast, trypsin or proteinase K treatment resulted in the generation of Uman antibody positivity with both antibodies, while staining for RPCA-NF-L-ct was diminished (Figs 7C, D, G and H). We also dissected spinal cord tissues from an untreated mouse and left them at room temperature for 4 h after sacrifice. The material left at room temperature contained numerous Uman-positive linear objects including striking beaded and sinusoidal profiles, while the staining for RPCA-NF-L-ct was attenuated (Fig. 6B). In contrast, control tissues fixed immediately post-mortem showed essentially no Uman-positive material. Both experiments support our hypothesis that proteases expose the Uman epitopes concomitantly with the destruction or wash out of NF-L tail epitopes.

### Generation and preliminary characterization of novel degeneration-specific antibodies

We raised antibodies against a proprietary recombinant construct including both Uman epitopes and some flanking sequence, amino acids 311-362 (Fig. 1B). As expected, both Uman antibodies and MCA-1B11 reacted strongly with this construct, consistent with our conclusions concerning the location of the epitopes for these three reagents (Fig. 2A, third blot lanes). Further characterization revealed clones that could be classified into at least four different groups, dubbed Classes I–IV. Class I includes UD1 and MCA-1B11 that were antibodies that bound to 316-370 immobilized in ELISA wells, bound less well to 316-357 and were only partially inhibited from binding to NF-L by the 25-amino acid–nested peptides. Class II antibodies included UD2 and several novel mouse monoclonal reagents including MCA-1D44. These showed strong binding to NF-L 316-370, 305-345, 316-357, 316-345 and more marginal or no binding for other peptides. These findings suggest that these new antibodies bind epitopes similar to or identical to that of UD2. Class III antibodies such as MCA-6H63 bind to the N-terminal of the 311-362 immunogen but show no binding to other peptides including the 316-370 peptide. Their epitopes are therefore heavily dependent on NF-L 305-310, distinct from both UD1 and UD2 (Fig. 1B). Class IV antibodies such as antibody 6D112 bound to all peptides including the 341-356 sequence. The results of peptide competition experiments for MCA-1D44, MCA-1B11 and MCA-6H63 are shown Supplementary Fig. 4. We also generated affinity purified rabbit and chicken polyclonal antibodies to the 311-362 peptide which we named RPCA-NF-L-Degen and CPCA-NF-L-Degen, respectively.

### Preliminary immunostaining results with novel antibodies

**Figure 8:**
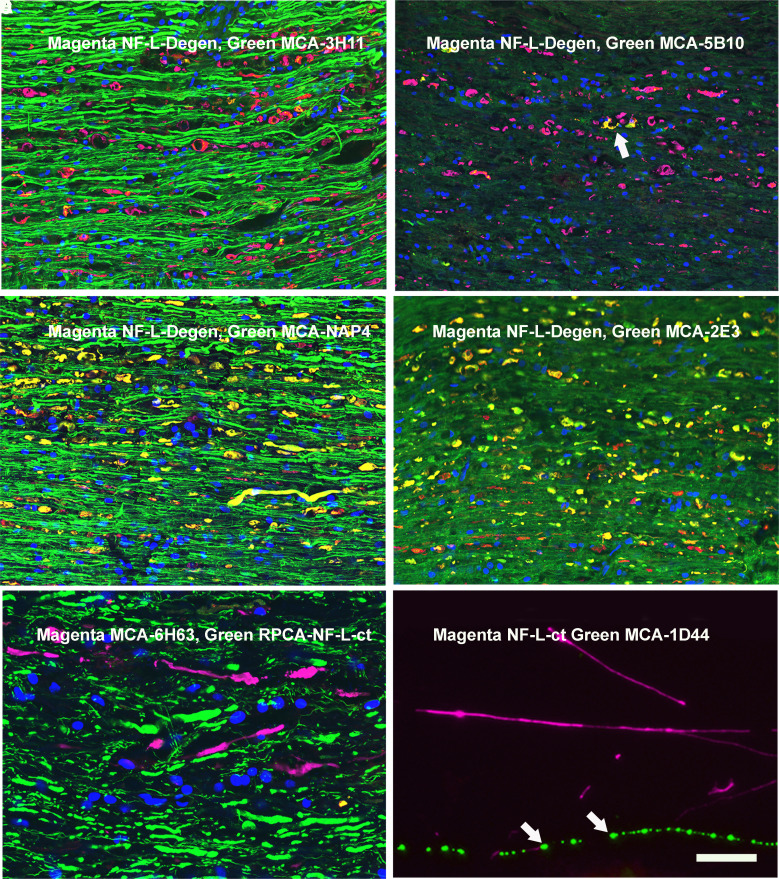
**Immunocytochemical studies with novel antibodies. A–D** are sections of spinal cord from a rat given a contusion 3 days previously and stained with RPCA-NF-L-Degen in magenta, and respectively in green: (**A**) the NF-M tail antibody MCA-3H11 that shows little staining of the Uman-positive aggregates. (**B**) The MAP-τ core domain antibody MCA-5B10 shows that MAP-τ associating with some inclusions (arrowed example) but apparently not others. (**C**) The phospho-KSP pNF-H antibody MCA-NAP4 strongly recognizes normal axons and the Uman-positive material that therefore appears yellow. (**D**) The α-internexin antibody MCA-2E3 shows that a part of this protein is also component of the Uman-positive material. **E** shows a longitudinal section of rat SCI tissue stained with MCA-6H63 in magenta and co-stained with RPCA-NF-L-ct in green. Despite having an epitope distinct from both UD1 and UD2, the MCA-6H63 antibody also clearly stains degenerating material and not healthy processes. **F** shows MCA-1D44 staining of mixed E20 cortical neural cultures in green co-stained with RPCA-NF-L-ct in magenta. As with the Uman reagents (Fig. 3C), MCA-1D44 fails to stain what are apparently healthy neuronal processes but does stain linear sets of aggregates (arrowed) which we conclude originated from degenerated processes. The blue signal in **A**–**E** is nuclear DNA revealed with DAPI. Bar in image **H** = 20 µM, applicable to all images.

The Class I monoclonal antibody MCA-1B11, the Class II MCA-1D44 and Class III MCA-6H63 all worked well on western blots and in peptide-binding studies and replicated the degeneration-specific immunofluorescense staining pattern seen with Uman reagents (see Fig. 6B and E for MCA-1B11; Figs 6F and 8E for MCA-6H63; and Fig. 8F for MCA-1D44). In our original studies, we noted a few very rare Uman antibody-positive fibres in spinal cord and brain sections of uninjured control animals (Fig. 3D–F). The availability of a panel of monoclonal antibodies with distinctly different epitopes allowed us to readdress this issue. We directly labelled the Class I monoclonal antibody MCA-1B11 with Alexa Fluor® 488 and the Class III monoclonal antibody MCA-6H63 with Alexa Fluor® 647. Control longitudinal sections of healthy spinal cord tissues revealed a few profiles that were negative for RPCA-NF-L-ct but were recognized strongly by both antibodies with an identical staining pattern, building confidence that these profiles are unlikely to be staining artefacts (Fig. 6D–F).

The novel rabbit and chicken polyclonal antibodies raised against the 311-362 peptide and also showed strong staining only of degenerating and degenerated material in spinal cord injury (SCI) cords (Fig. 8A–D for RPCA-NF-L-Degen). Given the availability of these reagents, we were able to identify degeneration-induced aggregates which we could then co-stain with various monoclonal antibodies providing a novel way of probing the axonal degradome. MCA-3H11, directed against a sequence in the C-terminal tail of NF-M,^31^ showed strong axonal staining like RPCA-NF-L-ct in green but only weak staining of the inclusions identified with RPCA-NF-L-Degen in magenta (Fig. 8A). This suggests that the NF-M tail epitope, like the comparable region of NF-L, is either destroyed by proteolysis or washes away on degeneration. Figure 8B shows staining for MAP-τ with MCA-5B10 that binds a peptide in the MAP-τ core domain (green) counterstained with RPCA-NF-L-Degen in magenta. Surprisingly, some aggregates stain strongly for MAP-τ, while others do not. Possibly MAP-τ washes out of the aggregates more quickly that the NF-L fragments. Figure 8C shows a similar section stained with MCA-NAP4 in green and RPCA-NF-L-Degen in magenta. MCA-NAP4 reacts with the phosphorylated lysine–serine–proline (KSP) repeats of pNF-H^32^ and strongly reacts with the RPCA-NF-L-Degen–positive inclusions. Apparently, this region of pNF-H does not readily wash out of the degenerated material. Figure 8D shows staining for MCA-2E3 antibody to α-internexin, which shows variable staining of the inclusions. The epitope for this antibody lies within the C-terminal 166 amino acids of rat α-internexin equivalent to the C-terminal 160 amino acids of the human molecule,^33^ corresponding to the tail region and the C-terminal part of Coil II. Future studies will follow up on these findings.

Figure 8E shows longitudinal rat SCI tissue stained with Class III antibody MCA-6H63 in magenta and counterstained with RPCA-NF-L-ct in green. Despite having an epitope N-terminal to and distinct from those of UD1 and UD2, the MCA-6H63 antibody also clearly specifically stains degenerating processes. Figure 8F shows Class II MCA-1D44 staining of mixed neural cultures in green counterstained with RPCA-NF-L-ct in magenta. As with the Uman reagents (Fig. 3C), MCA-1D44 fails to stain what are apparently healthy neuronal profiles but does stain linear sets of aggregates which we conclude originated from degenerated processes.

## Discussion

There has been an exponential increase in research reports describing the results of NF-L detection as a surrogate biomarker of axonal and neuronal loss in a variety of human and animal neurodegenerative states, ∼1000 publications appearing in the last 2 years. Accordingly, there should be considerable interest in our detailed characterization of the key reagents in the most widely used NF-L assays. Our findings give a better understanding of what exactly the Uman reagents are detecting, and we also describe novel immunoreagents with similar properties. Our work allows characterization of the various proteolytic NF-L fragments detected in CSF and blood associated with various damage and disease states^34–39^; see reviews by Yuan and Nixon^5^ and Petzold.^40^ Our data also suggest that the Uman epitopic region is proteolytically stable (Figs 6B and 7), and the numerous charged residues likely result in great solubility (Fig. 1B), both features desirable in a biomarker. Finally, Uman-type antibodies only detect degeneration-induced and likely extracellular forms of NF-L, an interesting and possibly beneficial property for a biomarker of neurodegeneration.

The data presented contain one very surprising finding that Uman-type antibodies do not bind NFs in healthy tissues that were strongly positive with other well-characterized NF antibodies (Figs 3–8). Clearly, they are highly specific for NF-L as evidenced by their convincing binding to native mammalian and recombinant NF-L on western blots (Fig. 2) and by direct and competitive peptide-binding experiments (Supplementary Figs 1–3). We propose that during degeneration, proteolysis of NFs is induced and the Uman and related epitopes become exposed and available for antibody binding. The concomitant loss of reactivity with several NF-L tail antibodies indicates that multiple tail epitopes of NF-L are either degraded or removed. The Uman reagents and the novel antibodies described here therefore uniquely allow the identification of degenerated and degenerating neuronal profiles, while NF-L tail antibodies allow the simultaneous visualization of healthy neuronal profiles. Neuronal profiles positive with both NF-L tail and Uman-type antibodies have presumably been caught undergoing degeneration. Our hypothesis is strengthened by our finding that Uman-type epitopes can be readily revealed by experimental protease treatment of tissue sections from healthy animals with no neural injury (Fig. 7) or by allowing tissues to naturally degrade (Fig. 6B). Our standard cell and tissue staining procedures generally do not reveal Uman-type epitopes on undamaged cells and processes. However, we have not tested antigen retrieval procedures that might partially denature NFs and so reveal previously hidden Uman-type epitopes.

The central region of the 316-370 peptide contains Stutter 2, a stretch of 11 amino acids that do not fit the typical hydrophobic heptad repeat pattern, and the UD1 epitope includes this region (Fig. 1B). Structural data on the highly homologous region of the closely related protein vimentin reveal a continuous α-helical coiled coil, the stutter merely introducing a slight kink.^15^ In agreement, both the AlphaFold2 and RoseTTAFold structural modelling algorithms predict that amino acids 316-357 of NF-L are part of a continuous elongated α-helix.^41,42^ The UD1 antibody could only be weakly inhibited from binding to NF-L by a subset of 25-amino acid peptides tested, and longer constructs such as 316-357 showed reduced inhibition when compared with the even longer 316-370 (Supplementary Fig. 2C). This suggests that UD1 likely binds to an outside face of the α-helical coiled-coil structure, interacting with a series of amino acids that are not adjacent in the primary sequence. UD2 in contrast has a more linear epitope since amino acids 318-330 effectively but not fully inhibit UD2 binding to NF-L (Supplementary Fig. 2D). Experimental addition of three appropriate amino acids to ‘repair’ the Stutter 1 hendecad sequence in vimentin to form two heptad repeats produces a construct that forms dimeric and multimeric α-helical coiled-coil assemblies.^38^ However, this construct does not further assemble into 10 nm filaments suggesting that this region has an important role in higher order filament assembly. 10 nm filament subunit sequences contain many instances where an amino acid at position i in an α-helical coiled coil is followed by one of opposite charge at position i + 4.^39^ The entire human NF-L Coil 2 sequence contains 145 amino acids and 15 of the i, i + 4 type sequences. Strikingly five highly conserved examples are in the 21 amino acids of NF-L 323-344 central to the Uman epitopic region (Fig. 1B). Letai and Fuchs^39^ suggested that such charge pairs form stabilizing intra-molecular interactions but may also be important in directing the formation of larger multimeric structures. Clearly, NF assembly must be directed by specific protein–protein interfaces between smaller protein complexes. We speculate that the Uman epitopic region is part of a binding site involved in NF assembly inaccessible in assembled NF but which is revealed during degeneration.

The Uman-type reagents have great utility beyond ELISA type assays. It will be interesting to apply them to the numerous existing animal models associated with axonal loss such as amyotrophic lateral sclerosis, multiple sclerosis, traumatic CNS injury and many others. They may also be useful reagents to identify spontaneous neuronal death in healthy animals and for tracing damaged fibres following surgical or chemical lesioning. The NF-L epitopes we have elucidated are defined by amino acid sequences that are 100% identical in apparently all mammals, so Uman-type antibodies will be applicable to studies of any mammalian species or disease model. Our work also shows that Uman-type antibodies bind peptides and *Escherichia coli* derived from recombinant proteins neither of which have mammalian post-translational modifications (PTMs). Possibly certain human damage and disease-associated NF fragments may have specific PTMs that alter the binding of Uman-type reagents, a topic for further study.

We note that Das *et al*. ^43^ recently described two novel NF-L monoclonal antibodies, ADx206 and ADx209, for use in NF-L ELISAs. The ADx209 epitope was mapped to LQELEDKQNAD, amino acids 333-343, which corresponds to the Stutter 2 region, making this a Class I antibody (Fig. 1B). The ADx206 epitope was mapped to VRAAKDEVSESRRL, amino acids 298-311, a few N-terminal amino acids to the epitope of our Class III antibody MCA-6H63. We also note that Budelier *et al*.^44^ also recently described novel NF-L antibodies, one of which, Hj30.4, also bound to the centre of Coil 2. It will be interesting to see if these antibodies share the degeneration-specific staining pattern described here.

## Conclusion

This study throws considerable light on exactly what the most widely used assays of neurodegeneration are detecting. They also raise many interesting questions. Which proteases generate this NF-L fragment, and where exactly do they cleave the NF-L molecule? Are different NF-L fragments generated in necrotic, apoptotic or other kinds of cell death? Does the hidden epitopic region have any function in activating inflammation or perhaps in appropriate circumstances regeneration? What is the half-life of the Uman-positive material following degeneration, and how does it get to the blood? If our speculation is correct and the Uman epitopic region is a binding site important for higher order filament assembly, what does it bind to? How does this method of visualizing neurodegeneration compare with other methods for the study of neurodegeneration, such as TUNEL, Fluoro-Jade and others? In conclusion, there are clearly numerous avenues for further investigation.

## Supplementary Material

fcad067_Supplementary_Data

## Data Availability

All original and relevant data generated as part of this study are available from the authors following reasonable request.

## Abbreviations

ATTC =: American Type Culture Collection
BNPS-Skatole =: 3-bromo-3-methyl-2-((2-nitrophenyl)thio)-3*H*-indole
BSA =: bovine serum albumin
C-terminal =: carboxy terminal
DAPI =: 4′,6-diamidino-2-phenylindole
GFAP =: glial fibrillary acidic protein
HGNC =: Human Genome Nomenclature Committee
His-tag =: hexahistidine tag
HPLC =: high-performance liquid chromatography
NF =: neurofilament
NF-L =: neurofilament light polypeptide
NTCB =: 2-nitro-5-thiocyanobenzoate
N-terminal =: amino terminal
pNF-H =: phosphorylated neurofilament heavy polypeptide
PTM =: post-translational modification
SCI =: spinal cord injury
S-tag =: ribonuclease S-protein tag
TBSt =: Tris-buffered saline plus 0.1% Tween 20
a-IFA =: anti-intermediate filament antigen
