# Supplementary material for: Uman-type neurofilament light antibodies are effective reagents for the imaging of neurodegeneration
Source: Brain Commun. 2023 Mar 16;5(2):fcad067. doi: 10.1093/braincomms/fcad067 (PMC10120172; doi:10.1093/braincomms/fcad067)
Supplement: fcad067_Supplementary_Data [file fcad067_Supplementary_Data.pdf]

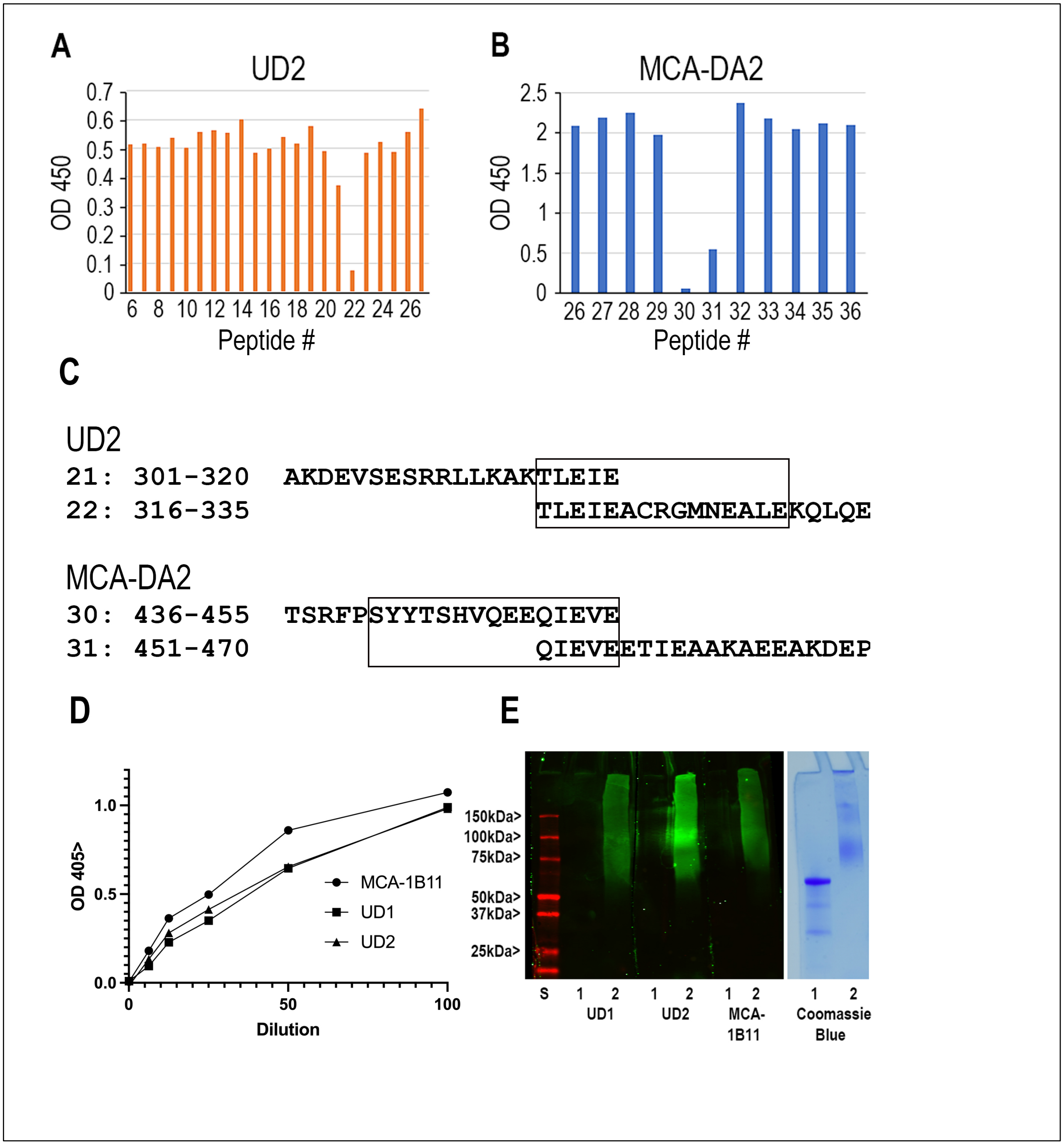

Supplementary Figure 1: Peptide inhibition and binding assays. A shows results of inhibition of binding of UD2 to full length recombinant NF-L by a set of 20 amino acid peptides each overlapping the next by 5 amino acids, each bar representing a single determination. Peptide 22 shows strong inhibition while peptide 21 shows some lesser inhibition. **B** shows results of the same experiment with MCA-DA2,

showing strong inhibition by peptide 30 and to a lesser degree by peptide 31. **C** summarizes the results diagrammatically, with the deduced core of the two epitopes boxed. These experiments provided no useful information for several other NF-L antibodies including notably UD1 and MCA-1B11. **D** shows the results of incubating serial dilutions of UD1, UD2 and MCA-1B11 on the NF-L 316-370 peptide coupled to ovalbumin and immobilized on an ELISA dish each data point representing a single determination. All three antibodies show strong and dilutable binding to the peptide conjugate while ovalbumin alone produced no signal. **E**, left panel shows western blotting of UD1, UD2 and MCA-1B11 on, in each lane 1, 4μg BSA and in each lane 2, 4μg BSA-coupled to NF-L 316-370. All three antibodies strongly bind a smear of high molecular weight material and do not stain BSA. Right panel shows a Coomassie blue stained protein gel of the same material, showing the high molecular weight smear in the BSA conjugate lane. Lane marked S shows molecular weight standards of the indicated size.

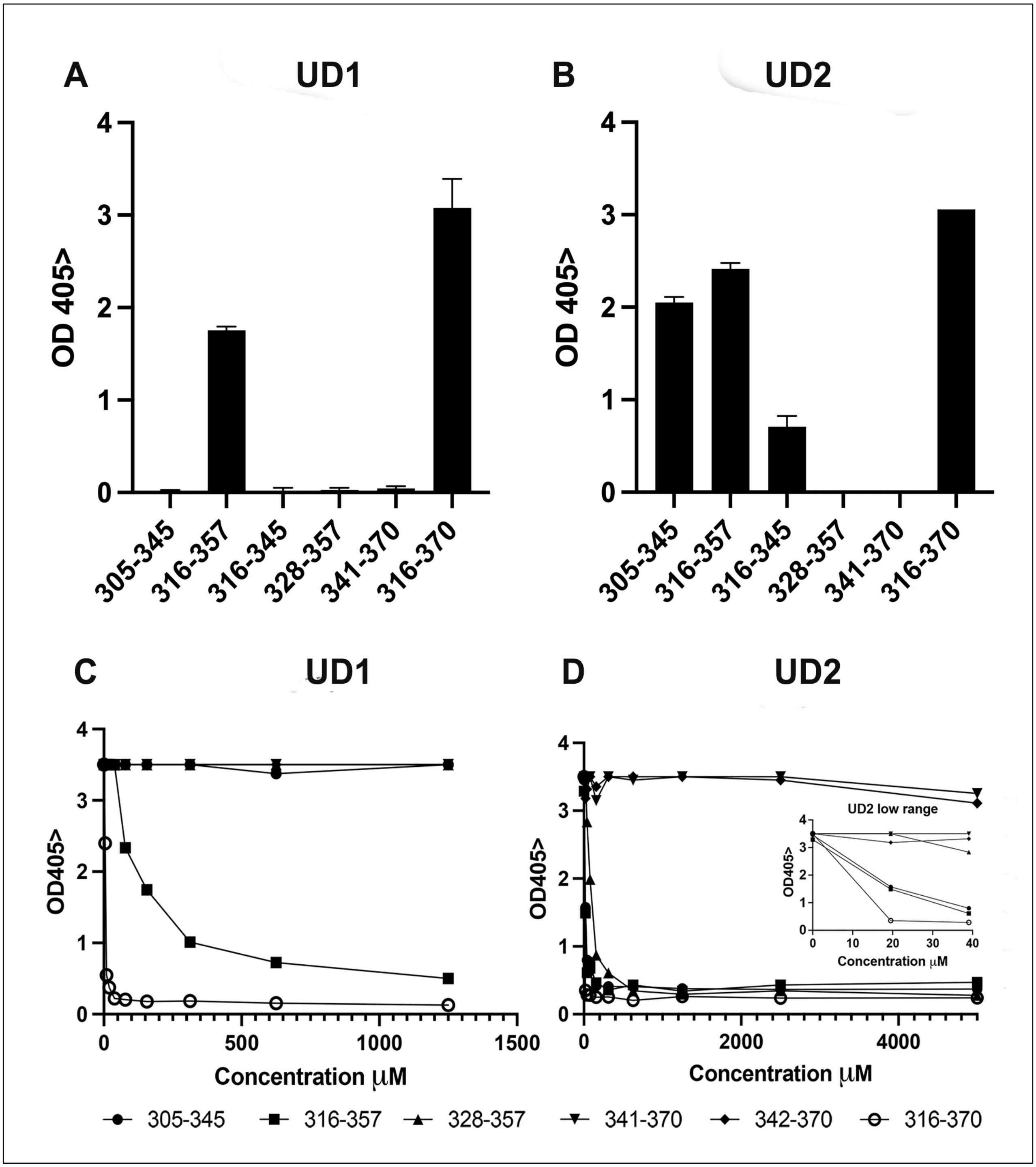

Supplementary Figure 2: Direct peptide binding and peptide competition experiments. The results of testing UD1 and UD2 binding to NF-L peptides 305-345, 316-357, 316-345, 328-357 and 341-370 (see **Fig. 1B**) applied in triplicate and equimolar amounts to ELISA plates. **A:** UD1 binds 316-370 and

less well to 316-357, with marginal binding to the other peptides. **B:** In the same assay UD2 binds 316-370 and less well to 305-345, 316-357 and 316-345, focusing attention on the shared 316-335 sequence in line with other data. Error bars show standard deviations. In **C, D** We challenged the binding of UD1 and UD2 to full length recombinant human NF-L in an ELISA format with a range of equimolar concentrations of the 6 peptides. In both cases and in line with direct binding data the 316-370 peptide was a very efficient inhibitor of antibody binding for both antibodies to NF-L. **C:** For UD1 316-357 showed less efficient but reproducible inhibition at the range of concentrations tested. **D:** In the case of UD2 all peptides containing the 316-335 sequence showed significant but less potent inhibition compared to the 316-370 peptide. Inset in **D** shows inhibition of UD2 binding at very low peptide concentrations. Each data point shows a single determination.

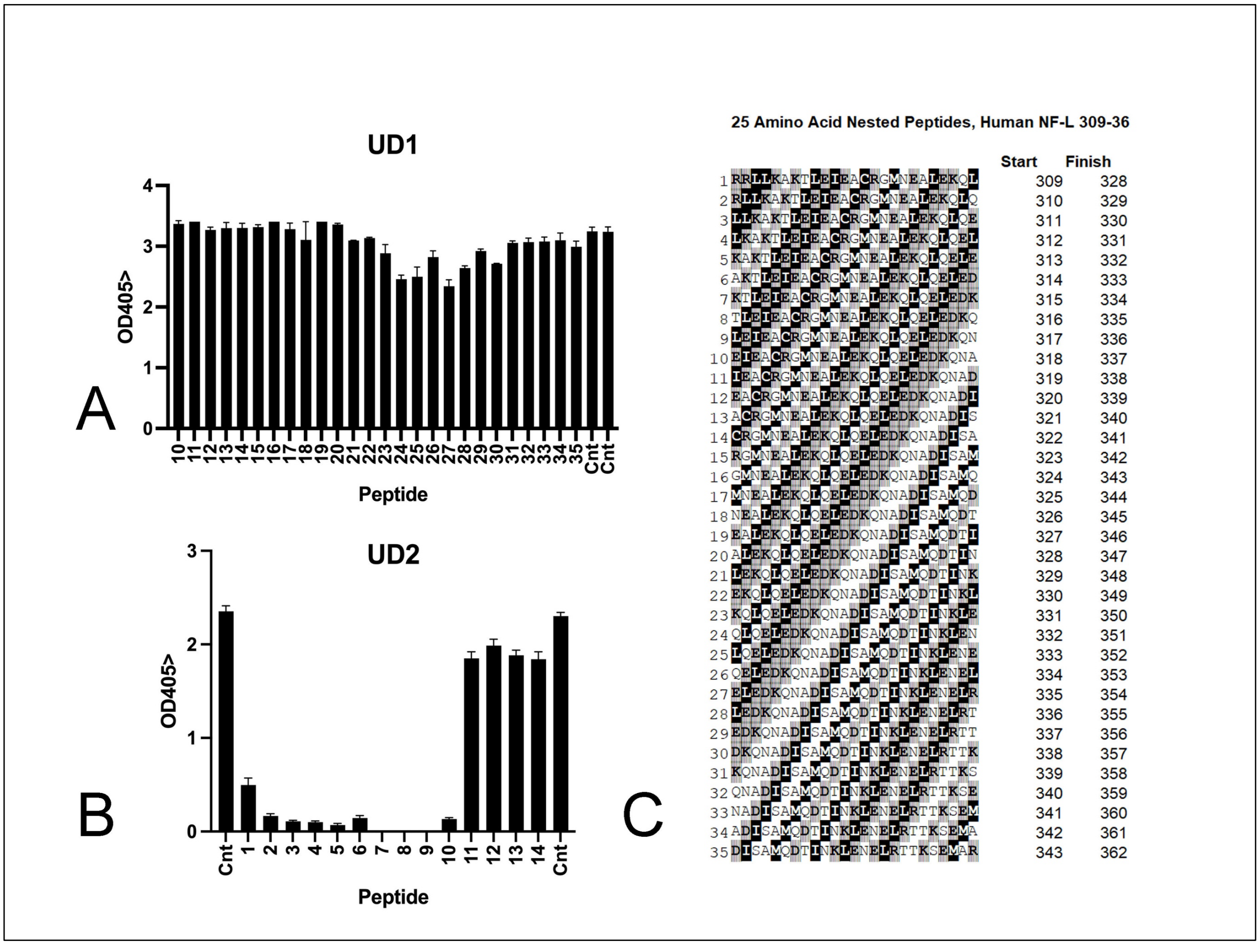

Supplementary Figure 3: Mapping of UD1 and UD2 epitopes by competition with 25 amino acid peptides each overlapping the next by one amino acid. Bars labelled “Cnt” were incubated with 2μL of 50% acetonitrile/50% distilled water in 100μL TBSt with the appropriate antibody and no peptide. UD1 is inhibited weakly by peptides 23-30, with the most efficacious being peptide 27. In contrast UD2 is strongly inhibited by peptides 1-10, with peptides 7-9 showing complete inhibition. Each plot shows average of two determinations and error bars show standard deviation. C shows full sequence of all peptides employed, with hydrophobic amino acids blocked out and charged amino acids cross-hatched.

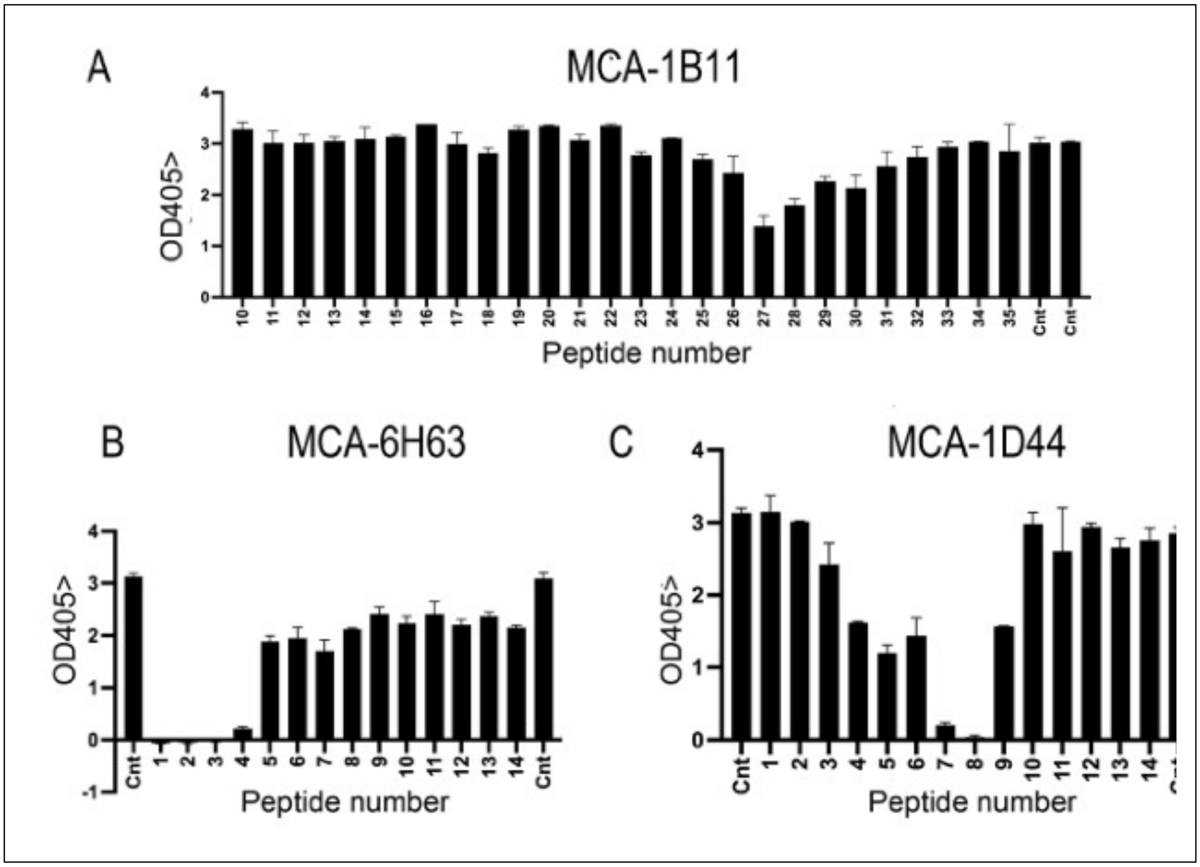

Supplementary Figure 4: Mapping of novel monoclonal antibodies MCA-6H63, MCA-1D44 and MCA-1B11 by 25 amino acid peptide inhibition. Bars labelled "Cnt" were incubated with 2µL of 50% acetonitrile/50% distilled water in 100µL TBSt with the appropriate antibody, all others show incubation of the indicated antibody with 2µL of 50% acetonitrile/50% distilled water plus the indicated peptide. MCA-6H63 is strongly inhibited by the first 4 peptides but shows more marginal inhibition by peptides 5-14, MCA-1D44 is strongly inhibited by peptide 8 and neighboring peptides, while MCA-1B11 is partially inhibited by peptide 27 and neighbors. MCA-1B11 is similar in binding properties to UD1, while MCA-1D44 is very similar in specificity to UD2. MCA-6H63 therefore has an epitope distinct from UD1, UD2, MCA-1B11 and MCA-1D44. Each plot shows average of two determinations and error bars show standard deviations. The peptides used and experimental procedures were identical to those utilized in supplementary figure 3C.

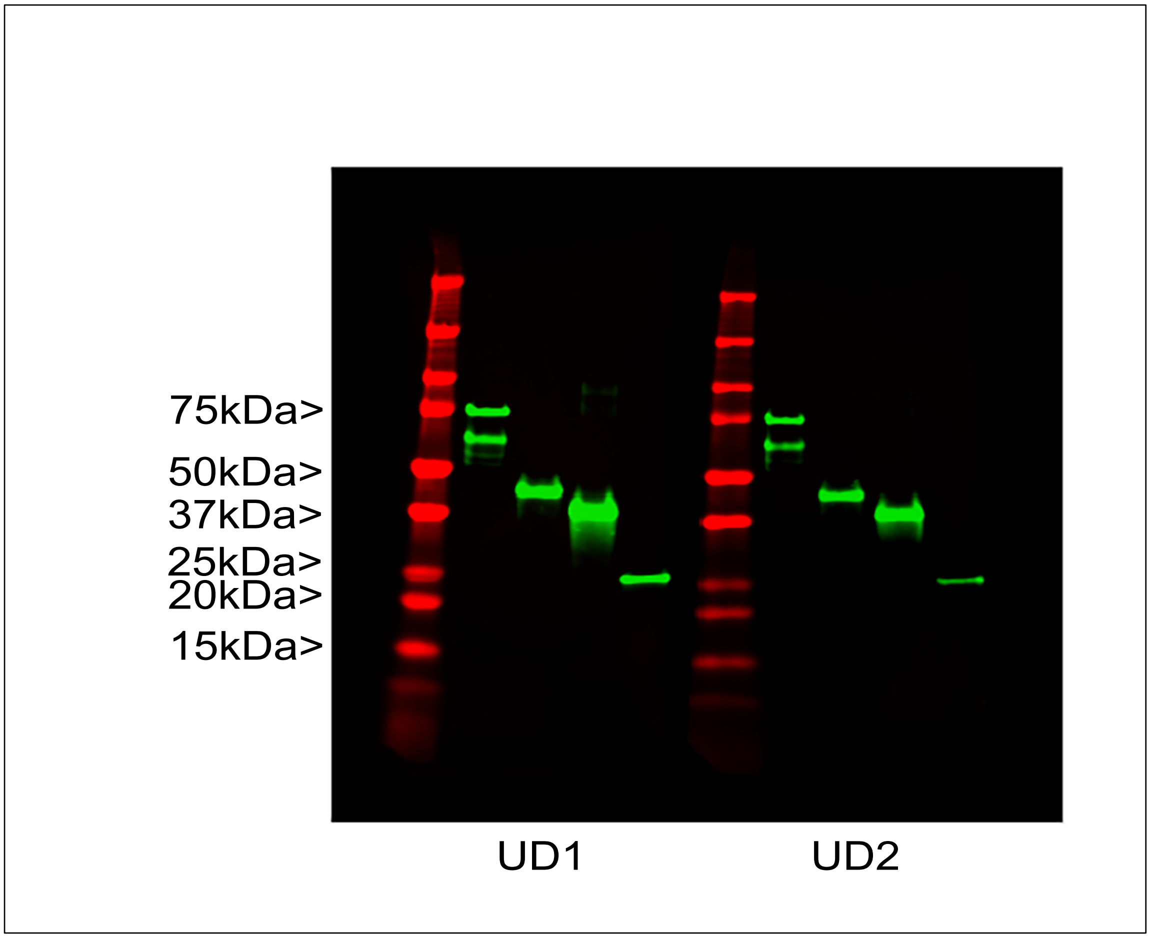

Supplementary Figure 5: Original western blot for two left panels of **Fig. 2A**.

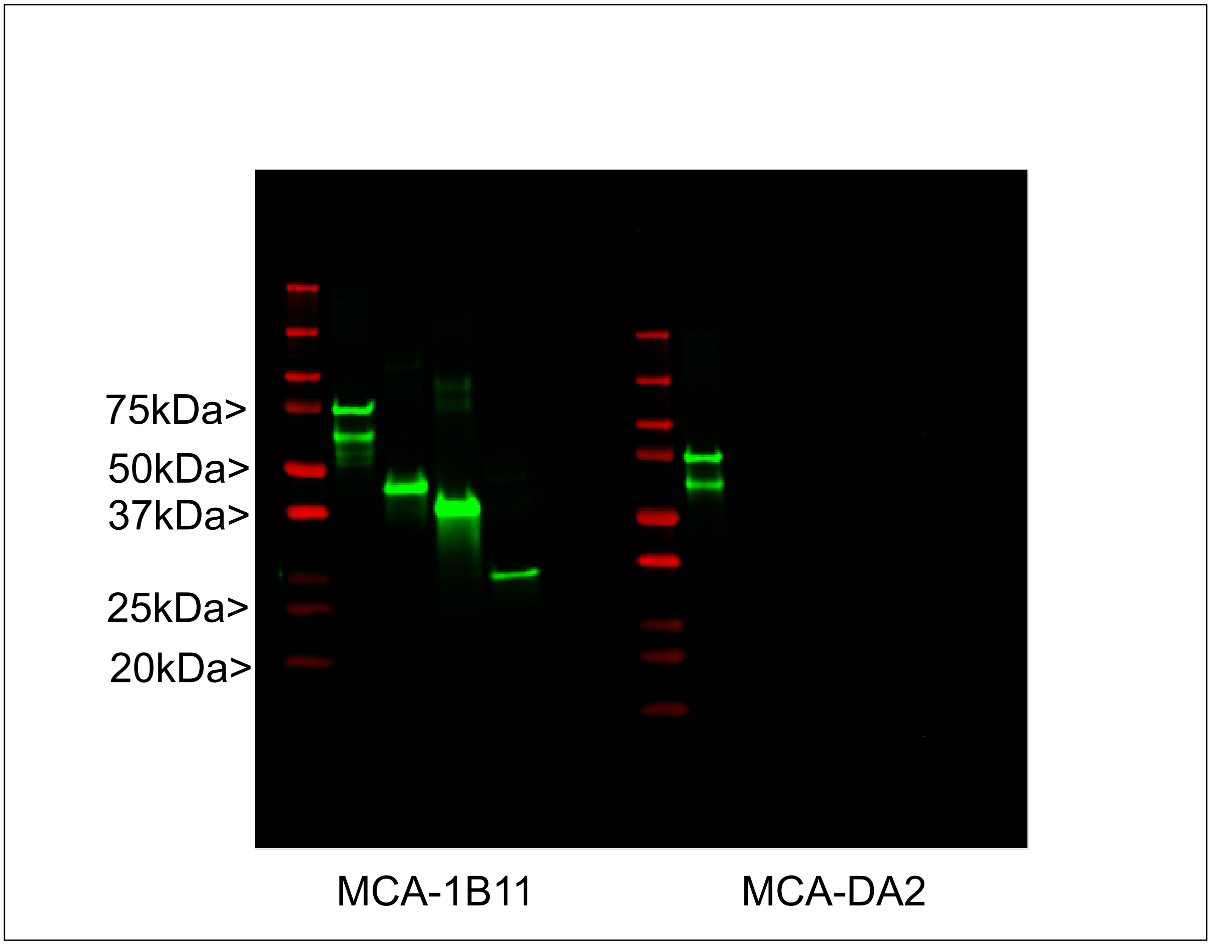

Supplementary Figure 6: Original western blot for right panel of **Fig. 2A**, MCA-DA2 data not used in this publication.

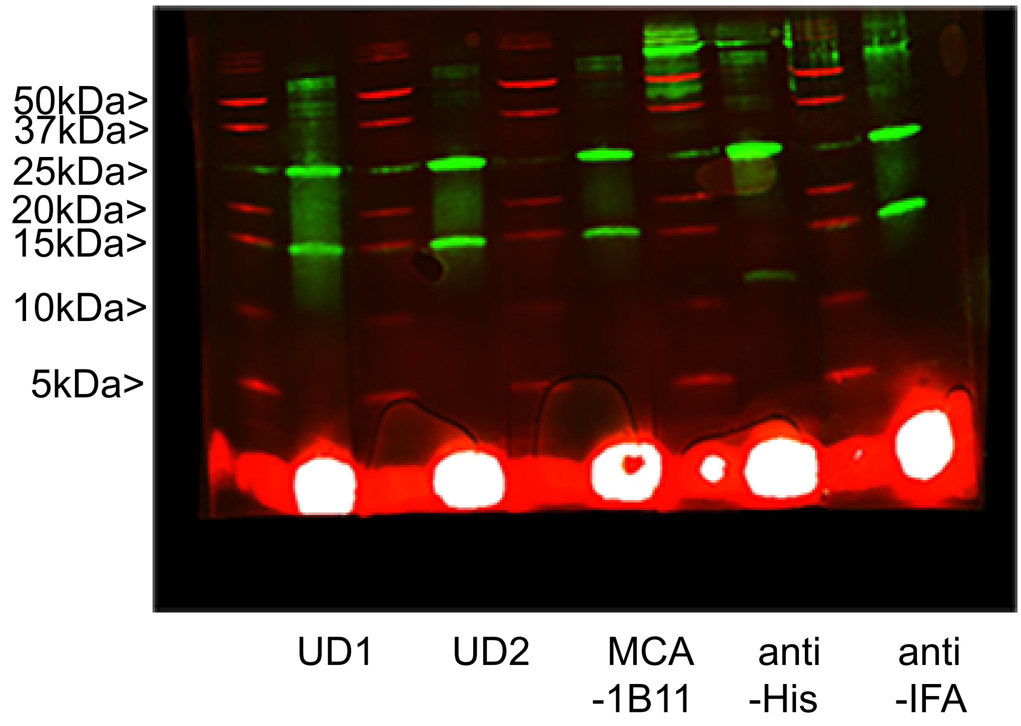

Supplementary Figure 7: Original western blot of **Fig. 2B**.

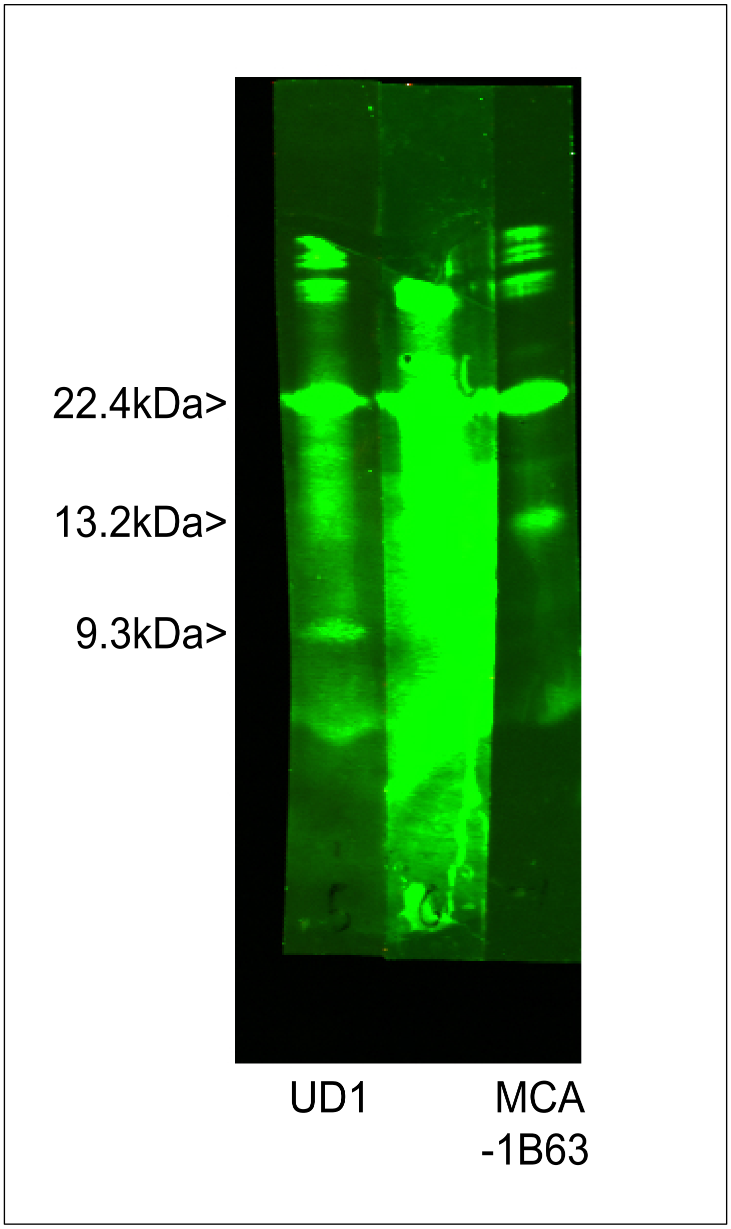

Supplementary Figure 8: Original western blot of first and fourth lanes of **Fig. 2C.**

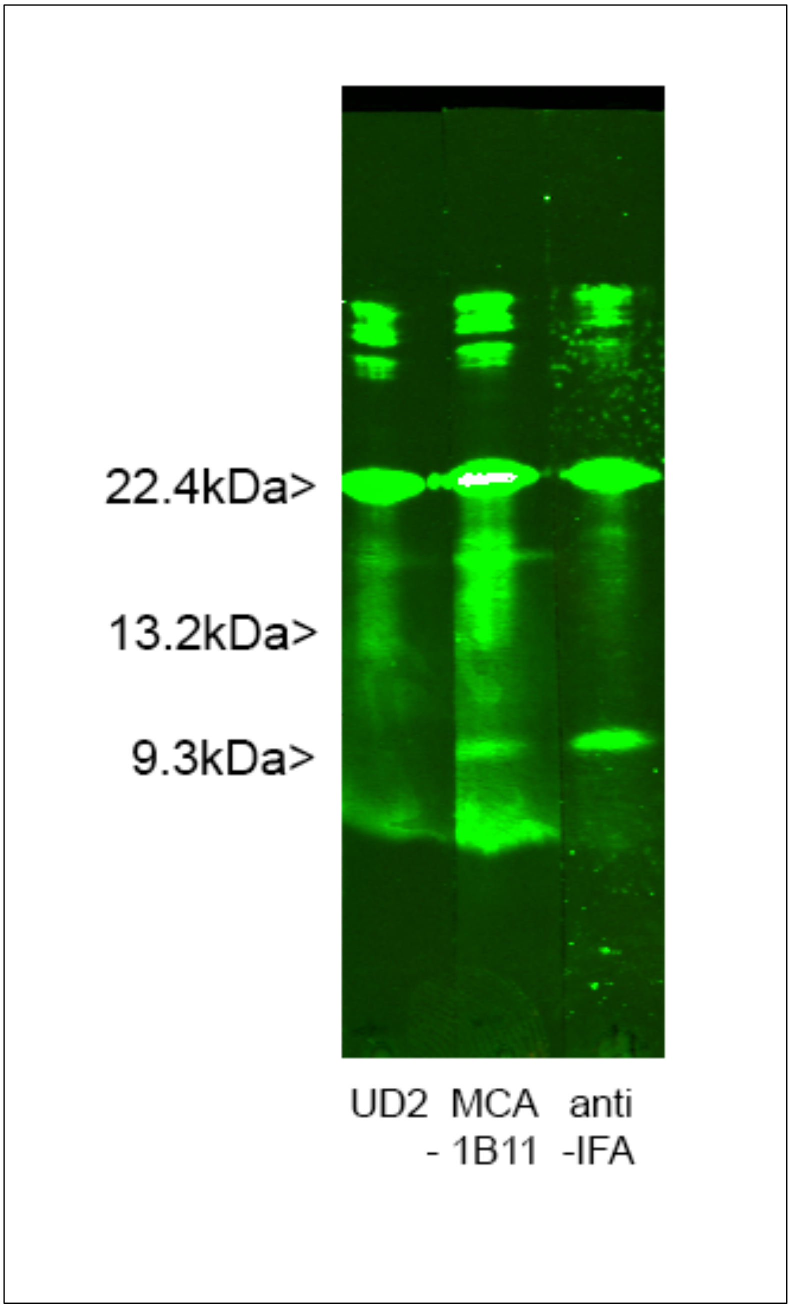

Supplementary Figure 9: Original western blot of second, third and fifth lanes of F**ig. 2C**.

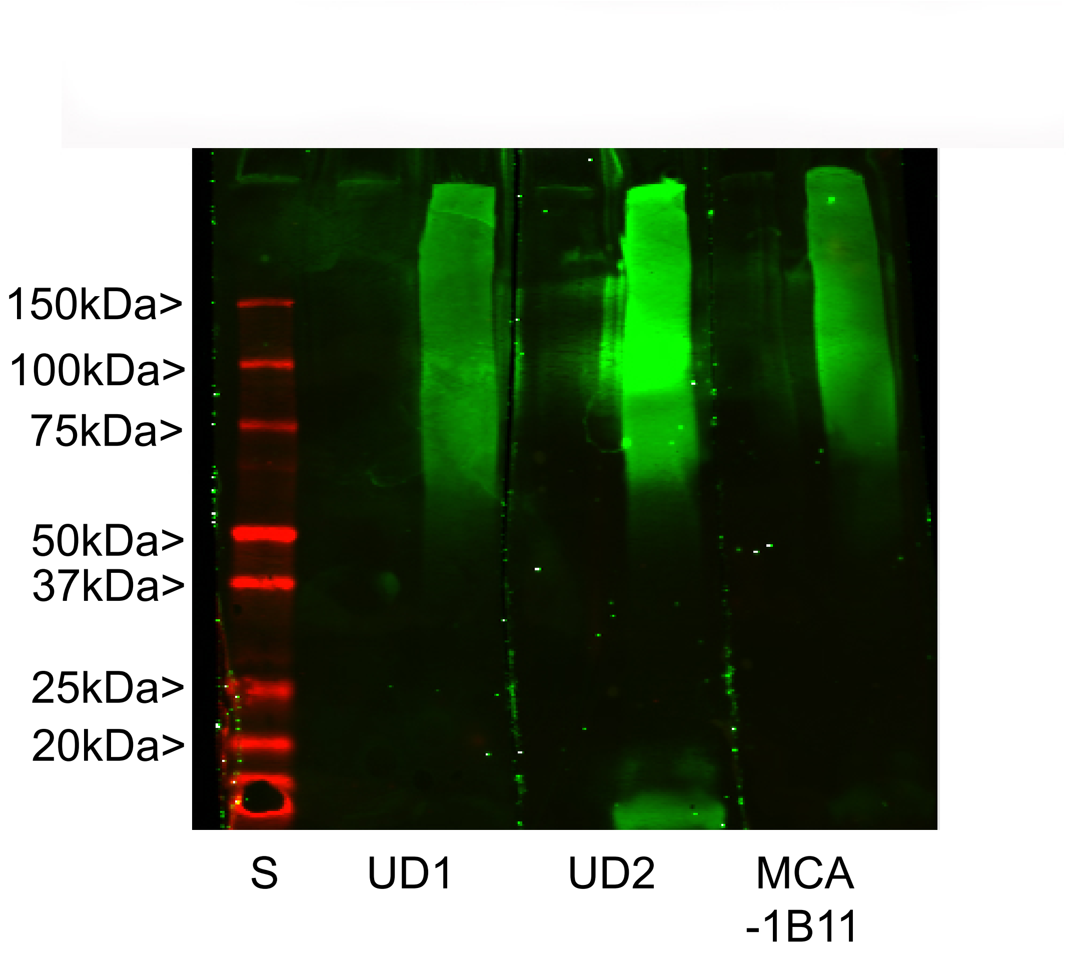

Supplementary Figure 10: Original western blot of left three lanes, bottom panel of **Supplementary Figure 1.**

Supplementary Table 1

Name and link	Source	Immunogen	Specificity
UD1/2.1	Uman	HPLC purified mammalian spinal cord NF-L	Human NF-L 322-357
UD2/47.3	Uman	HPLC purified mammalian spinal cord NF-L	Human NF-L 316-330
MCA-DA2	EnCor	Pig neurofilament preparation	Human NF-L 441-455
MCA-6H63	EnCor	Immunogen including NF-L 311-362	Human NF-L 311-315
MCA-1D44	EnCor	Immunogen including NF-L 311-362	Human NF-L 316-330
MCA-1B11	EnCor	Purified pig spinal cord NF-L	Human NF-L 322-357
MCA-6H112	EnCor	Human NF-L C-terminal peptide	Human NF-L 514-542
MCA-6D112	EnCor	Immunogen including NF-L 311-362	Human NF-L 341-356
MCA-3H11	EnCor	Recombinant rat NF-M tail	Rat NF-M tail 762-845
MCA-2E3	EnCor	Recombinant rat α-internexin	Human α-internexin 340-505
MCA-1B63	EnCor	Recombinant pET30a(+) leader sequence	S-tag peptide
MCA-NAP4	EnCor	NF-H purified from bovine spinal cord	NF-H phospho-KSP sequences
MCA-5B10	EnCor	Human low molecular weight MAPτ	Tau isoform hTau40 362-381
RPCA-NF-L-ct	EnCor	C-terminal 29 amino acids of rat NF-L	Rat NF-L C-terminus 515-543
RPCA-NF-L-Degen	EnCor	Recombinant human NF-L 311-362	Human NF-L 311-362
CPCA-NF-L-Degen	EnCor	Recombinant human NF-L 311-362	Human NF-L 311-362
Anti-IFA/TIB-131	ATCC	GFAP from human spinal cord	Human NF-L 381-400
Ab18184	Abcam	Poly histidine peptide	6 sequential histidine residues
4F8	Giasson lab	phospho-Ser 473 NF-L peptide 465-480	Phospho-Ser 473 NF-L 465-480

All antibodies listed are mouse monoclonals with the exception of polyclonals RPCA-NF-L-ct and RPCA-NF-L-Degen, which were made in rabbit and polyclonal CPCA-NF-L-Degen, which was made in chicken.
